# Identifying PDAP1 as a Biological Target on Human Longevity: Integration of Mendelian Randomization, Cohort, and Cell Experiments Validation Study

**DOI:** 10.1111/acel.70065

**Published:** 2025-04-10

**Authors:** Tianzhichao Hou, Zimo Sha, Qi Wang, Yuanyue Zhu, Zheng Zhu, Huajie Dai, Yijie Zhu, Tiange Wang, Mian Li, Zhiyun Zhao, Yu Xu, Jieli Lu, Jie Zheng, Jing Ye, Weiqing Wang, Guang Ning, Yufang Bi, Weiguo Hu, Min Xu

**Affiliations:** ^1^ Department of Endocrine and Metabolic Diseases, Shanghai Institute of Endocrine and Metabolic Diseases, Geriatric Medical Center, Ruijin Hospital Shanghai Jiao Tong University School of Medicine Shanghai China; ^2^ Shanghai National Clinical Research Center for Metabolic Diseases, Key Laboratory for Endocrine and Metabolic Diseases of the National Health Commission of the PR China, Shanghai Key Laboratory for Endocrine Tumor, State Key Laboratory of Medical Genomics, Ruijin Hospital Shanghai Jiao Tong University School of Medicine Shanghai China; ^3^ Geriatric Department, Geriatric Medical Center, Ruijin Hospital Shanghai Jiao Tong University School of Medicine Shanghai China; ^4^ Department of Cardiology, Ruijin Hospital Shanghai Jiao Tong University School of Medicine Shanghai China; ^5^ MRC Integrative Epidemiology Unit, Bristol Medical School University of Bristol Bristol UK; ^6^ Department of Surgery, Ruijin Hospital Shanghai Jiao Tong University School of Medicine Shanghai China

**Keywords:** drug targets, longevity, mediation analysis, mendelian randomization, PDAP1, proteomics, tissue‐specific analysis, transcriptomics

## Abstract

Identifying factors affecting lifespan, including genes or proteins, enables effective interventions. We prioritized potential drug targets and provided insights into biological pathways for healthy longevity by integrating Mendelian randomization, cohort, and experimental studies. We identified causal effects of tissue‐specific genetic transcripts and serum protein levels on three longevity outcomes: the parental lifespan, the top 1% and 10% extreme longevity, utilizing Mendelian randomization and multi‐traits colocalization, combining the latest genetics data of gene expression (eQTLGen and GTEx) and proteomics (4746 proteins from five studies). We then evaluated associations of these potential genetic targets with mortality risk and life expectancy in the UK Biobank cohort. We performed in vitro cellular senescence experiments to confirm their effects. Fourteen plasma proteins and nine transcripts in whole blood had independent causal effects on longevity, where a cascading effect of both the tissue‐specific transcripts and plasma proteins of LPA, PDAP1, DNAJA4, and TMEM106B showed negative effects on longevity. PDAP1 (PDGFA‐associated protein 1) with the strongest genetic evidence might reduce lifespan by modifying sex hormones, adiposity, and epigenetic aging acceleration. In the prospective cohort, blood PDAP1 levels were significantly associated with higher all‐cause mortality and more years of loss. In vitro, cellular senescence is accompanied by upregulation of PDAP1 expression. Exogenous PDAP1 stimulation accelerates cellular senescence while the deficiency of PDAP1 attenuates replicative senescence. This study facilitates the discovery of potential drug targets and provides a broader understanding of the biological processes of longevity, where PDAP1 emerged as a star for modifying human lifespan.

## Graphical abstract

Integration of multi‐omics analyses reveals PDAP1 as one of the key regulators of longevity and aging. Mendelian randomization, colocalization, prospective studies, and in vitro experiments highlight PDAP1's effects on mortality, lifespan, and cellular senescence.

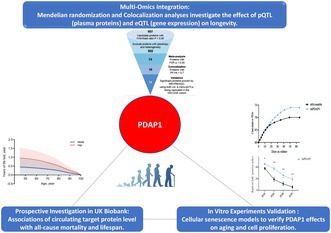

## 1 Introduction

The increasing incidence of age‐related diseases worldwide, such as cardiovascular diseases, cancers, and neurodegeneration, imposes a substantial decrease in human lifespan (Campisi et al. 2019; Guo et al. 2022; López‐Otín et al. 2023). Conventional medical research predominantly focuses on organ‐specific diseases, disregarding the pathogenic processes of the entire human body that impact lifespan. Therefore, identifying factors affecting human lifespan and longevity, including genes or proteins, enables targeting effective interventions. Such investigations would empower the development of strategies to mitigate age‐related diseases, ultimately increasing lifespan and promoting longevity.

Research on aging mechanisms has presented new prospects for interventions in extending longevity (Campisi et al. 2019; Jensson et al. 2023) including from genetics. Large genome‐wide association studies (GWAS) have uncovered genetic loci associated with human lifespan (Deelen et al. 2019; Pilling et al. 2016, 2017; Timmers et al. 2019; Zeng et al. 2016), especially among those with extremely long lives (Sebastiani et al. 2012). These studies have yielded the genetic variations that may contribute to extended lifespan and offered valuable insights into the genetic architecture of longevity. Combined with the genetic data, the molecular quantitative trait loci (QTLs) significantly contribute to understanding human longevity at a molecular level (Kaushik and Cuervo 2015; Tyshkovskiy et al. 2023). These multi‐omics analyses have become increasingly popular for drug target identification (Chen et al. 2022a; Gaziano et al. 2021; Jamie et al. 2022; Zheng et al. 2022) and driven genes investigation (Kia et al. 2021; Mavromatis et al. 2023; Xu et al. 2022). Nevertheless, the existing targets related to longevity and lifespan reported in OpenTargets (Ochoa et al. 2023) and Drugbank (Wishart et al. 2018) are scarce, primarily due to insufficient clinical trial evidence, which poses a challenge to the identification and prioritization of drugs.

Previous studies have identified potential aging biomarkers using omics data analysis. However, a comprehensive understanding of the interconnected biological pathways that integrate gene expression (eQTLs) and protein (pQTLs) with tissue‐specific functionality is not fully understood yet (Mavromatis et al. 2023; Perrot et al. 2021; Timmers et al. 2022). Therefore, we employed an integrative approach that combines genomics, transcriptomics, proteomics, and phenomics to identify tissue‐specific actionable targets, linking genetic transcription and translation with human longevity‐related outcomes (including lifespan and extreme longevity outcomes) based on Mendelian randomization (MR) and colocalization. We then verified our genetic findings using in vitro cellular senescence models and cohort association analysis. This interdisciplinary approach facilitates the discovery of potential drug targets and provides a broader understanding of the intricate biological processes associated with longevity.

## 2 Results

**FIGURE 1:**
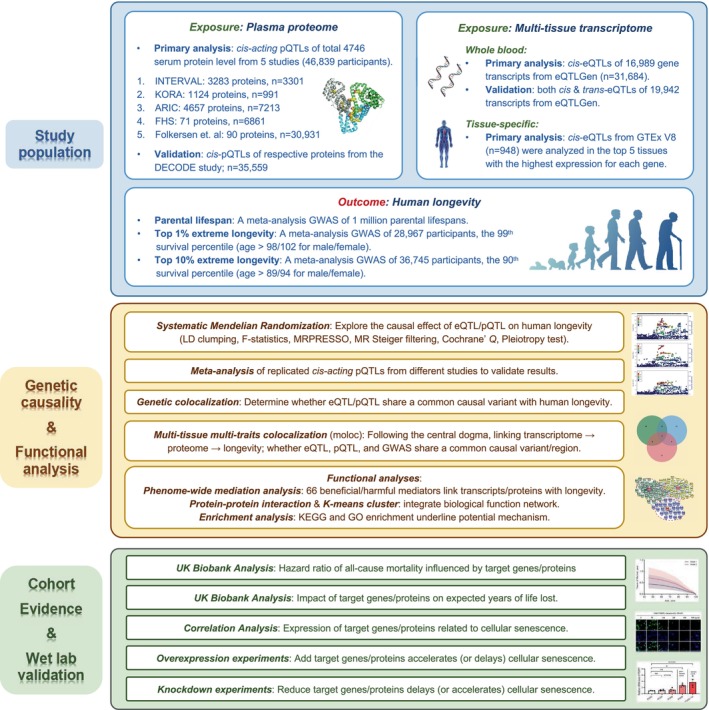
Study design and the flowchart. The present study included three stages. First, we selected the study population and determined exposures and outcomes. Two sets of exposures: The serum protein levels (pQTLs) and the genetic expression levels in multi‐tissues (eQTLs); three longevity‐related traits of outcomes: The parental lifespan (years), the top 1% and top 10% extreme longevity were included. Second, causality for eQTL and pQTL on human longevity outcomes was established using Mendelian randomization (MR), colocalization, and meta‐analysis with several sensitivity analyses. The molecular function and involved pathways were investigated using phenome‐wide MR scanning with mediation MR analysis, protein–protein interaction with cluster analysis, KEGG & GO enrichment, and literature mining. Finally, for any prioritized targets, we used different cellular senescence models in vitro to solidify their function and investigated the targets as biomarkers in the independent UK Biobank cohort. ARIC, Atherosclerosis Risk in Communities; eQTL, Expression quantitative trait loci; FHS, Framingham Heart Study; GWAS, Genome‐wide association study; KORA, Kooperative Gesundheitsforschung in der Region Augsburg; moloc, Multi‐traits colocalization; pQTL, Protein quantitative trait loci.

Figure 1 describes the overall scheme of the study design. We used plasma protein levels (pQTLs) and multi‐tissue gene expression levels (eQTLs) as exposures, and three longevity‐related traits as outcomes: the parental lifespan (continuous trait, years), the top 1% and top 10% extreme longevity (case–control) (Deelen et al. 2019; Timmers et al. 2019). The data sources are in Table S1. In the first step, we estimated the putative causal effects of the genetic targets on longevity outcomes using MR and multi‐traits colocalization. In the second step, we explored the underlying mechanisms among the genetic targets generated from the first step by conducting enrichment analysis, protein–protein interaction (PPI), K‐means cluster, and a phenome‐wide MR analysis with subsequent mediation analysis. Finally, we verified the biological link between potential targets and longevity using the cellular senescence model in vitro and investigated the targets as potential biomarkers of risk of mortality and expected lifespan in the UK Biobank.

### 2.1 Instruments of Plasma Protein (pQTLs) and Tissue‐Specific Gene Expressions (eQTLs) Level

We used a previously well‐defined pipeline to generate genetic instruments for pQTLs and eQTLs of potential genetic targets (Zheng et al. 2022). Detail was described the Method part. We extracted pQTLs of plasma protein levels from five genetic studies and eQTLs of whole blood from the eQTLGen while tissue‐specific eQTLs from the GTEx V8 (GTEx Consortium 2020; Liu et al. 2022; Võsa et al. 2021).

### 2.2 Longevity Outcomes and Their Genetic Correlation

We selected three human longevity outcomes: lifespan of 1,012,240 parental survivors from a large meta‐GWAS analysis, top 1% extreme longevity (28,967 participants) and top 10% extreme longevity (36,745 participants) from two meta‐GWAS analyses of 18 case–control studies (Deelen et al. 2019; Timmers et al. 2019). We estimated the genetic correlation among the three outcomes using bivariate LD score regression. The genetic correlations between lifespan and the top 1%/10% extreme longevity were 0.65 and 0.81, respectively; the genetic correlation between top 1% and 10% extreme longevity was 1.01 (all *p* < 0.001). This indicated specific differences in the genetic background between lifespan and extreme longevity outcomes (Table S2).

### 2.3 Putative Causal Effects of the Plasma Proteome on Human Longevity

**FIGURE 2:**
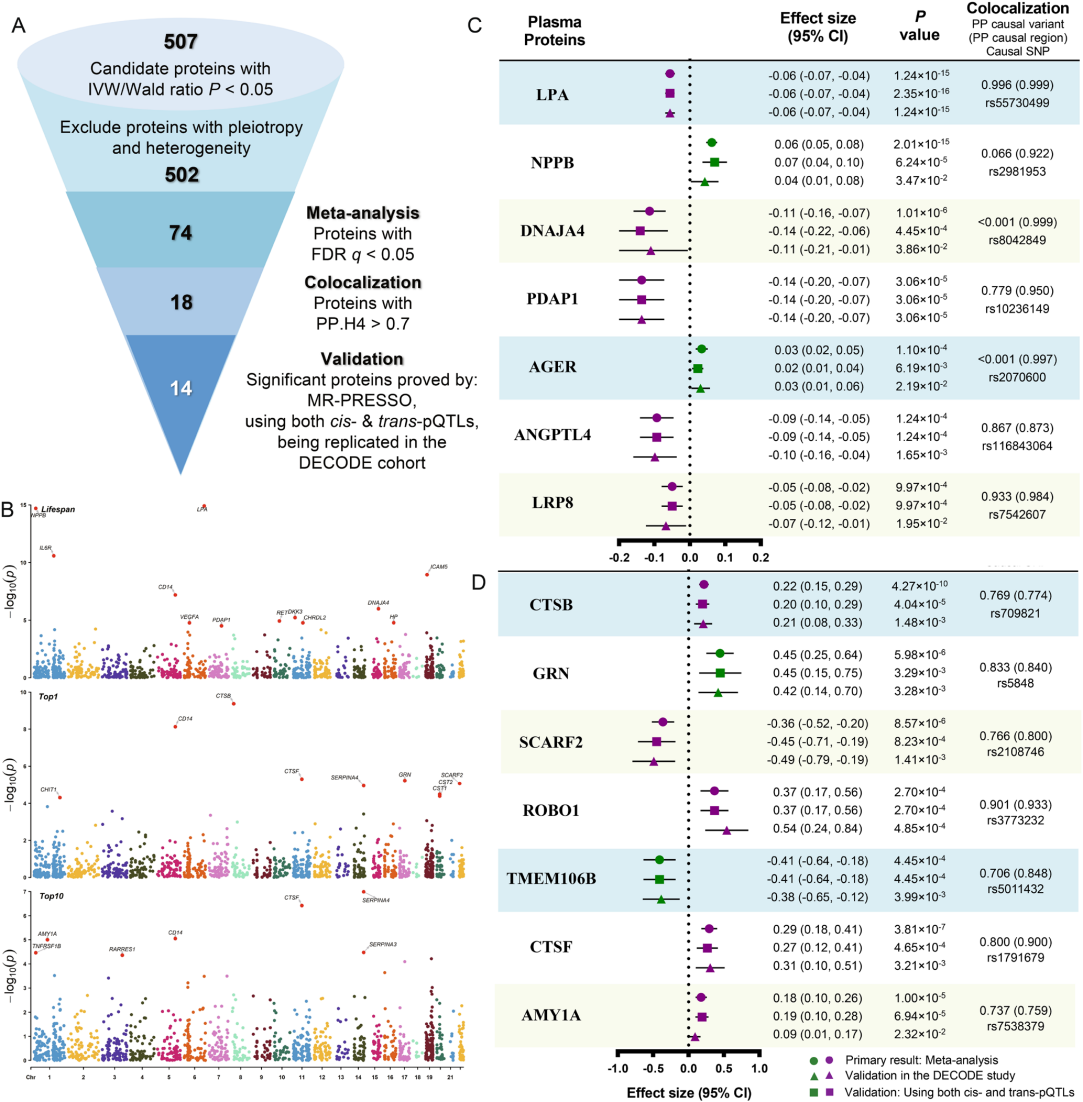
Integrating significant plasma proteins on human longevity outcomes. It integrates MR results after meta‐analysis with colocalization evidence of the causal effect of plasma proteins on human longevity outcomes. (A) The funnel plot indicates our steps for the filtration. First, we excluded any candidate proteins with pleiotropy and heterogeneity from the original results (IVW/Wald ratio *p* < 0.05). Second, we performed the meta‐analysis based on a random‐effect model and selected proteins with FDR *q* < 0.05. Third, proteins with colocalization PP.H4 > 70% remained. Finally, the remaining proteins validated by MR‐PRESSO, using both cis‐ and trans‐pQTLs, and being replicated in the DECODE cohort were included. (B) The Manhattan plot shows the distribution of significant individual proteins related to each outcome. (C) Seven plasma cis‐acting proteins with FDR‐corrected significant MR results and colocalized evidence (PP.H4 > 70%) on parental lifespan. (D) Plasma cis‐acting proteins with FDR‐corrected significant MR results and colocalized evidence (PP.H4 > 70%) on top 1% (CTSB, GRN, SCARF2, ROBO1, TMEM106B) and top 10% (CTSF, AMY1A) extreme longevity. AGER, Advanced Glycosylation End‐Product Receptor; AMY1A, Alpha‐amylase 1; ANGPTL4, Angiopoietin‐like 4; CTSB, Cathepsin B; CTSF, Cathepsin F; DNAJA4, Heat Shock Protein Family (Hsp40) Member A4; GRN, Granulin; LPA, Lipoprotein (a); LRP8, LDL receptor related protein 8; NPPB, Brain natriuretic peptide; PDAP1, Platelet‐derived growth factor subunit A‐associated protein 1; PP.H4, Posterior probability of hypothesis 4; ROBO1, Roundabout homolog 1; SCARF2, Scavenger receptor class F member 2; SE, Standard error; TMEM106B, Transmembrane protein 106B.

Given the potential pleiotropy and false positive rate, we used a very strict filtration pipeline to refine our results of MR analysis (Figure 2A). We meta‐analyzed all duplicated MR signals from different original proteomic studies as our main results (Figure 2B). We identified 48 plasma proteins that were significantly associated with lifespan under a false discovery rate (FDR) threshold (*p* < 1.67 × 10^−3^); 13 plasma proteins with the top 1% extreme longevity (*p* < 4.45 × 10^−4^); and 14 plasma proteins with the top 10% extreme longevity (*p* < 3.91 × 10^−4^) (Table S3). The pairwise z‐score comparison showed little difference in significant proteins among parental lifespan and top 1% / 10% extreme longevity (*p* = 0.93), where CD14 had a consistent genetically causal effect on all three longevity outcomes after FDR correction (Figure S1).

We finally defined putative causal effects that had a posterior probability of regional colocalization (PP.H3 + PP.H4) > 70% and were validated by using both cis‐ and trans‐acting pQTLs and were replicated in the independent DECODE cohort (Figure 2C,D). There are 7 plasma proteins that affect parental lifespan: LPA, NPPB, DNAJA4, PDAP1, AGER, ANGPTL4, and LRP8 (Figure 2C); 5 plasma proteins on the top 1% extreme longevity: CTSB, GRN, SCARF2, ROBO1, and TMEM106B; and 2 plasma proteins on the top 10% extreme longevity: CTSF and AMY1A (Figure 2D).

Based on the results from plasma proteins, we performed a series of functional analyses including the phenome‐wide mediation MR, protein–protein interaction (PPI), and GO and KEGG enrichment analyses. Integrating 3079 health‐related diseases or traits as potential mediating factors (Detail in Tables S4 and S5), the mediation MR added more evidence to the effect of plasma proteins on longevity outcomes. For instance, NT‐proBNP positively impacts cardiovascular diseases and type 2 diabetes, and improves lung function, which is beneficial for lifespan (Table S6). The PPI network and enrichment analysis also indicated that lifespan‐associated proteins could be grouped into three clusters according to their interactions using the K‐means cluster (Figure S2): neoplasm, immune response, and lipid metabolism. GO and KEGG enrichment of proteins in the three subgroups further supported the primary cluster results (Figure S3).

### 2.4 Putative Causal Effects of the Whole Blood Gene Expression on Human Longevity

Given that plasma protein levels are influenced by upstream gene expression, we next investigated whether whole‐blood gene transcripts exhibit similar causal effects on longevity outcomes. This analysis helps to bridge the gap between transcriptomic regulation and protein‐driven phenotypic effects, providing additional mechanistic insights into longevity‐associated pathways. For the whole‐blood transcripts, we finally identified *cis*‐acting genetic transcripts of 7 genes (*HYKK*, *NRG1*, *NTN5*, *BECN1*, *ADD1*, *PDAP1*, and *SRFBP1*) had a significant putative causal effect on parental lifespan after FDR correction (*p* < 1.35 × 10^−4^); 2 genes (*KALRN* and *RP11‐71H17.7*) had a significant causal effect on the top 1% extreme longevity (*p* < 4.31 × 10^−6^). However, no genes had significant causal effects on the top 10% extreme longevity (Table 1 and Table S7). All genes have a colocalization posterior probability of sharing a common causal variant (PP.H4) > 70%. The effect of *cis*‐acting eQTLs was further verified by using both cis‐ and trans‐acting eQTLs. Overlapped transcripts for the three outcomes were integrated in Figures S4 and S5.

**TABLE 1:** Replicable significant MR results of eQTLs on human longevity outcomes and related diseases or traits.

Gene (ENSG)	Effect of *cis*‐acting eQTLs				Effect of both *cis*‐ and *trans*‐acting eQTLs				Steiger test	Colocalization		Top 4 significantly related diseases or traits in phenome‐wide mediation MR analysis
	Effect size (SE)	*p*	*P*_pleio_	*P*_het_	Effect size (SE)	*p*	*P*_pleio_	*P*_het_		PP H4	Coloc SNP	
*Outcome: Parental Lifespan (continuous)*												
*HYKK* ENSG00000188266	0.769 (0.075)	6.26 × 10^−25^	NA	NA	0.562 (0.055)	6.26 × 10^−25^	NA	NA	TRUE	0.986	rs8042849	Lung cancer*, FEV1*, Dental problems, Cystatin C
*NRG1* ENSG00000157168	0.028 (0.005)	4.96 × 10^−8^	0.207	0.896	0.019 (0.004)	1.80 × 10^−5^	0.424	0.020	TRUE	0.838	rs2466076	Urate, Stroke*, Cystatin C, Blood pressure
*NTN5* ENSG00000142233	−0.083 (0.015)	5.35 × 10^−8^	0.644	0.645	−0.056 (0.011)	1.81 × 10^−7^	0.354	0.477	TRUE	0.940	rs569970	Cholesterol, Triglycerides*, Blood pressure, IBD
*BECN1* ENSG00000126581	−0.066 (0.012)	9.10 × 10^−8^	0.236	0.699	−0.043 (0.010)	1.30 × 10^−5^	0.306	0.246	TRUE	0.994	rs1011157	Waist circumference*, Breast cancer*, HbA1c*, HDL
*ADD1* ENSG00000087274	0.128 (0.024)	9.79 × 10^−8^	0.530	0.553	0.087 (0.020)	2.14 × 10^−5^	NA	0.246	TRUE	0.732	rs6819310	FEV1, Dental problems, Frailty index, Diabetes*
*PDAP1* ENSG00000106244	−0.308 (0.073)	2.45 × 10^−5^	NA	NA	−0.213 (0.050)	2.45 × 10^−5^	NA	NA	TRUE	0.787	rs10155966	Testosterone, SHBG, Waist circumference, Blood pressure
*SRFBP1* ENSG00000151304	0.260 (0.067)	9.51 × 10^−5^	NA	NA	0.179 (0.046)	9.51 × 10^−5^	NA	NA	TRUE	0.773	rs75965538	Blood pressure*, Stroke, Coronary artery disease
*Outcome: Top 1% extreme longevity (case–control)*												
*KALRN* ENSG00000160145	−0.811 (0.165)	8.28 × 10^−7^	0.350	0.532	−0.570 (0.130)	1.20 × 10^−5^	NA	0.535	TRUE	0.902	rs13069961	No significance
*RP11‐71H17.7* ENSG00000260391	−0.362 (0.079)	4.31 × 10^−6^	0.699	0.850	−0.667 (0.149)	7.71 × 10^−6^	NA	NA	TRUE	0.931	rs10804562	No significance

*Note:* All significant transcripts on human longevity outcomes after FDR correction in both primary analysis (*cis*‐eQTL only) and validated analysis (both cis and trans eQTLs) with strong colocalization evidence (PP. H4 > 0.7). NA indicates inadequate number of SNPs to perform the analysis. β and SE are the effect sizes with standard error of a single eQTL on the outcome. *p* value is the significance of the result of a single eQTL on the outcome. *P*_pleio_ is the pleiotropy based on MR‐Egger analysis. *P*_het_ is the heterogeneity using Cochran's Q test. Steiger test is the direction test of the result. PP (posterior probability) H4 is the posterior probability of the eQTL and the outcome shared the common causal variant that is the Coloc SNP column. The top 4 FDR‐corrected significant diseases or traits (FDR *q* < 0.05) were from the subsequent phenome‐wide mediation MR analysis, where * indicates the relationship between FDR‐corrected significant phenotypes (diseases or traits) and significant genes (eQTLs) has been reported in previous publications with reasonable direction.

Abbreviations: FEV1, Forced Expiratory Volume in 1 s; HDL, High‐density lipoprotein; IBD, Inflammatory bowel disease; SHBG, Sex hormone binding globulin.

The same phenome‐wide mediation MR and PPI functional analyses were also performed (Figure S6). We identified several diseases or traits that connected genetic transcripts to longevity outcomes (Table 1 and Table S8). For example, elevated *NTN5* transcript level was associated with a shorter parental lifespan, which was mediated by cholesterol, triglycerides, blood pressure, and inflammatory bowel disease.

### 2.5 Integration of the Plasma Proteome With the Tissue‐Specific Transcriptome

To ensure the concordance between plasma protein and their corresponding gene expression levels in addition to the whole blood, we examined the effect of tissue‐specific transcripts of significant proteins on longevity outcomes (Table 2 and Table S9). Plasma LPA corresponds with gene expression of *LPA* in the liver tissue. For tissue‐specific eQTLs, we selected genetic transcripts based on all 14 significant causal proteins for each longevity‐related outcome. The plasma proteins and respective tissue‐specific gene transcripts of *LPA* (liver), *PDAP1* (whole blood) and *DNAJA4* (thyroid and skin) had consistent causal effects on the parental lifespan; *TMEM106B* (skin) had a consistent causal effect on the top 1% extreme longevity. All results had a posterior probability for causal region > 70% in the multi‐tissue multi‐traits colocalization (*moloc*) analysis, where PDAP1 had the strongest posterior probability of sharing a causal variant across the gene transcript, plasma protein, and the parental lifespan.

**TABLE 2:** Tissue specific analysis connecting transcripts, plasma proteins, and longevity.

Gene	Tissue	MR Effect: cis‐pQTL/cis‐eQTL		Multi‐traits colocalization		Top 4 significantly related diseases or traits in phenome‐wide mediation MR analysis
		Effect size (95% CI)	*p*	PP causal variant	PP causal region	
*Outcome: Parental Lifespan (continuous variable)*						
*LPA*	Plasma protein	−0.06 (−0.07, −0.04)	1.24 × 10^−15^	/	/	Coronary artery disease*, Triglycerides*, Heart failure*, Blood pressure*
	Liver	−0.07 (−0.09, −0.05)	4.21 × 10^−10^	0.011	1.000	
*PDAP1*	Plasma protein	−0.11 (−0.16, −0.07)	1.01 × 10^−6^	/	/	SHBG, Testosterone, Waist circumference, Blood pressure, Heart failure, Basal metabolic rate
	Whole blood	−0.31 (−0.45, −0.16)	2.45 × 10^−5^	0.850	0.951	
*DNAJA4*	Plasma protein	−0.14 (−0.20, −0.07)	3.06 × 10^−5^	/	/	HbA1c, Years of schooling
	Thyroid	−0.04 (−0.07, −0.01)	4.23 × 10^−3^	< 0.001	0.999	
	Sun Exposed Skin	−0.05 (−0.08, −0.01)	4.99 × 10^−3^	< 0.001	0.999	
	Not Sun Exposed Skin	−0.05 (−0.08, −0.01)	4.99 × 10^−3^	< 0.001	0.999	
*Outcome: Top 1% extreme longevity (case–control)*						
*TMEM106B*	Plasma protein	−0.41 (−0.64, −0.18)	4.45 × 10^−4^	/	/	Alzheimer's disease*
	Sun Exposed Skin	−0.63 (−1.15, −0.11)	1.76 × 10^−2^	< 0.001	0.743	

*Note:* *p* value is the significance of the result of a single QTL on the outcome. PP (posterior probability) causal region is the posterior probability of the eQTL, pQTL, and outcomes shared one common causal region (*P*_a,b,c_ + *P*_a,bc_ + *P*_ab,c_ + *P*_ac,b_ + *P*_abc_) while PP causal variant is the posterior probability of the eQTL, pQTL, and outcomes shared one common causal variant (Pabc). The top 4 FDR‐corrected significant diseases or traits (FDR *q* < 0.05) were from the subsequent phenome‐wide mediation MR analysis, where * indicates the relationship between FDR‐corrected significant phenotypes (diseases or traits) and significant plasma proteins (pQTLs) has been reported in previous publications with reasonable direction.

Abbreviation: SHBG, Sex hormone binding globulin.

### 2.6 PDAP1 Had Negative Effects on Longevity via Multiple Potential Pathways

**FIGURE 3:**
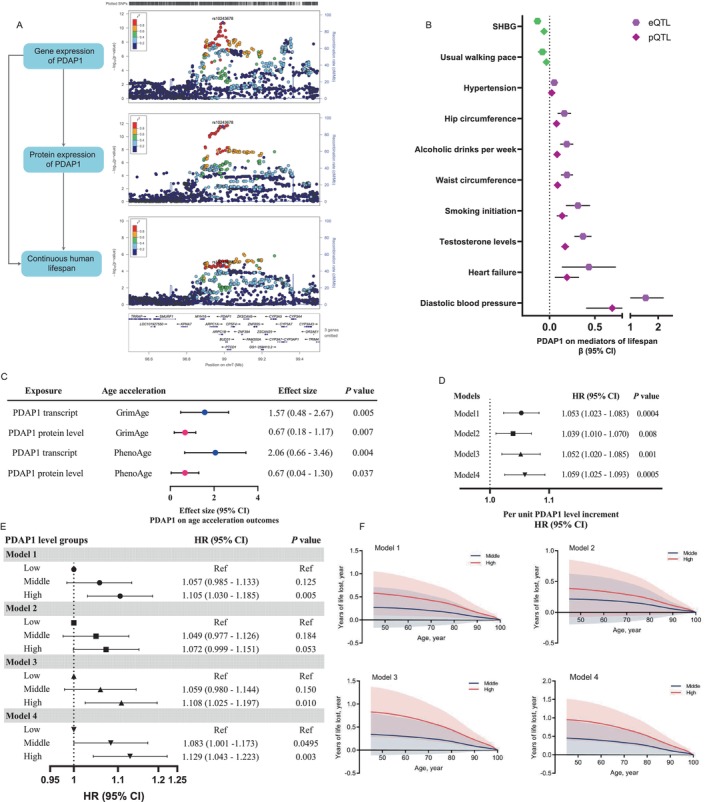
Functional investigation of plasma proteins and focus on the novel PDAP1 on longevity. (A) The moloc plot (*PDAP1* gene, ±500 kb) links genetic expression in whole blood, plasma protein level of PDAP1 to the human lifespan outcome following the central dogma: Both eQTL/pQTL (regardless of cis‐ or trans‐acting ones) of PDAP1 showed a significant effect on lifespan with high colocalization evidence. The variant rs10243678 was located as a coloc variant for eQTL, pQTL, and longevity outcome. (B) The effect of both gene transcripts and plasma protein of PDAP1 on mediators in relation to the outcome of lifespan. Mediators beneficial to lifespan are shown in green (SHBG and usual walking pace), and harmful to lifespan are shown in purple (other mediators). (C) The effect of both gene transcripts and plasma protein of PDAP1 on the acceleration of the aging process (PhenoAge and GrimAge). (D) The association between per unit level increased plasma PDAP1 level and the risk of all‐cause mortality. Model 1 adjusted for age and sex. Model 2 was further adjusted for diabetes status, smoking status, and body mass index. Model 3 further adjusted for systolic blood pressure, diastolic blood pressure, total cholesterol, triglycerides, high‐density lipoprotein cholesterol, and low‐density lipoprotein cholesterol. Model 4 was further adjusted for protein Lp (a) and DNAJA4 levels. (E) The all‐cause mortality influenced by plasma PDAP1. Plasma PDAP1 level was categorized into low, middle, and high levels based on tertile value. (F) Years of life lost associated with middle and high‐level PDAP1 when compared to the low PDAP1 group. PDAP1 level was categorized into low, middle, and high levels based on tertile value. The reference group is the low‐tertile group. SHBG, Sex‐hormone binding globulin.

Particularly, we observed robust evidence from MR and colocalization to support the causal effect of both transcript and protein levels of *PDAP1* on human longevity (Figure 3A). Multi‐traits colocalization analysis showed robust colocalization evidence for PDAP1 to share a common genetic causal variant (rs10243678, shared posterior probability = 85.0%) and a causal region (chr11:9185624–10274538, shared posterior probability = 95.1%) among the gene expression, plasma protein levels, and lifespan, strengthening the biological pathway according to the central dogma (Table 2 and Figure 3A).

Based on the phenome‐wide mediation MR analyses from the Sections 2.3 and 2.4, both the eQTL and pQTL of PDAP1, previously reported to be related to cancer, were associated with several cardiometabolic traits that reduce lifespan. The elevated level of genetic expression and plasma protein of PDAP1 was associated with decreased sex hormone binding globulin (SHBG) (β_pQTL_: −0.06 [−0.08, −0.05]; β_eQTL_: −0.13 [−0.18, −0.10]), increased testosterone levels (β_pQTL_: 0.17 [0.13, 0.21]; β_eQTL_: 0.37 [0.27, 0.46]), increased diastolic blood pressure (β_pQTL_: 0.69 [0.40, 0.98]; β_eQTL_: 1.55 [0.90, 2.20]), increased waist circumference (β_pQTL_: 0.09 [0.06, 0.12]; β_eQTL_: 0.19 [0.13, 0.26]), and heart failure (β_pQTL_: 0.19 [0.06, 0.32]; β_eQTL_: 0.43 [0.14, 0.73]) (all reached statistical significance after FDR *q* < 0.05) (Figure 3B, Tables S6 and S8). These suggested the elevation of PDAP1 might influence lifespan via metabolic effects in addition to cancer.

To further determine the aging mechanism of PDAP1, we performed an additional mediation MR analysis with epigenetic accelerators of aging as a potential mediating process. We included both GrimAge (trained on mortality, including a DNA methylation measure of smoking as a constituent part) and PhenoAge (a DNA methylation predictor trained on a measure that itself was trained on mortality, using 42 clinical measures and age as input features) as “epigenetic clocks” (McCartney et al. 2021). Both the eQTL and pQTL levels of PDAP1 were associated with higher levels of epigenetic aging acceleration (Figure 3C).

### 2.7 Circulating PDAP1 Level Associates With Higher Mortality in the UK Biobank

We evaluated the association of circulating PDAP1 levels with risk of mortality and expected life expectancy in the UK Biobank dataset. After adjustments for age, sex, body mass index, smoking, diabetes status, systolic and diastolic blood pressure, total cholesterol, high‐ and low‐cholesterol, triglycerides, Lp(a) and DNAJA4 levels (Model 4), circulating PDAP1 levels, both in each‐unit increase and the categorical analysis to a tertile change (high‐, middle‐ and low‐group), were significantly associated with a higher risk of all‐cause mortality (*p* = 0.01 and 0.003, respectively, Figure 3D,E and Table S10). Furthermore, life expectancy decreased as the level of PDAP1 increased. In participants aged 60 years high PDPA1 levels were associated with 0.84 years lost (95% confidence interval 0.34, 1.35), as compared to the low‐level group, after the same adjustment as above (Figure 3F).

### 2.8 Elevated PDAP1 Levels Induce Senescence and Reduce Cell Proliferation

**FIGURE 4:**
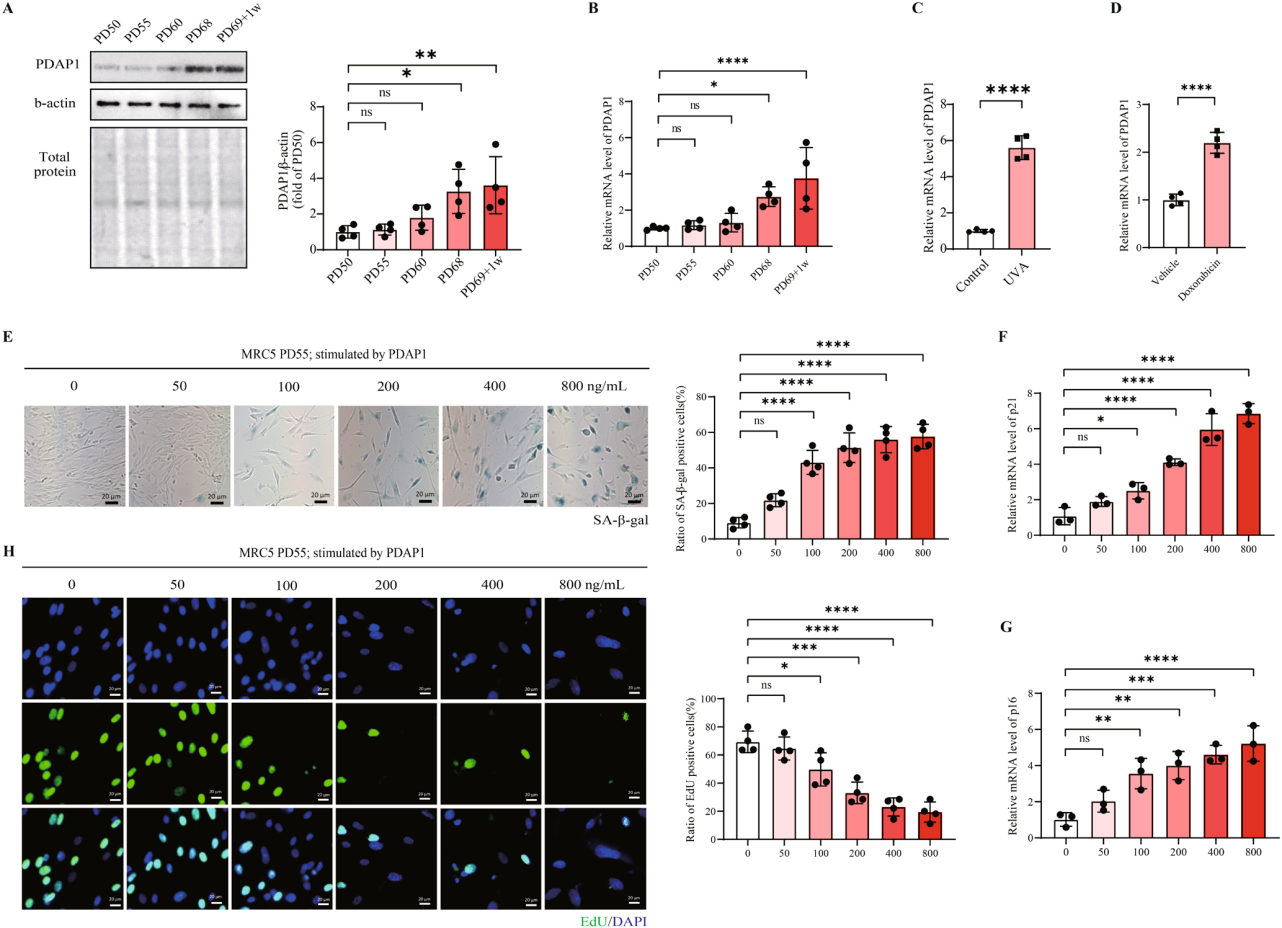
PDAP1 is upregulated under senescence and exogenous PDAP1 stimulation accelerates cellular senescence. (A) Representative immunoblotting images showing PDAP1 protein levels in MRC‐5 cells collected at different population doublings (PDs). Quantification was from four independent experiments. (B) Real‐time quantitative reverse transcription polymerase chain reaction analysis comparing PDAP1 mRNA expression in MRC‐5 cells at different PDs. (C and D) Real‐time quantitative reverse transcription polymerase chain reaction analysis comparing PDAP1 mRNA expression in MRC‐5 cells treated by UVA (C) or doxorubicin (D) and control groups. (E) Representative images showing the senescence‐associated β‐galactosidase in MRC‐5 cells at different concentrations of exogenous PDAP protein. Quantification was the percentage of cells positive for senescence‐associated β‐galactosidase. (F and G) Real‐time quantitative reverse transcription polymerase chain reaction analysis comparing p16 (F) and p21 (G) mRNA expression in MRC‐5 cells at different concentrations of exogenous PDAP protein. (H) Representative images showing the EdU in MRC‐5 cells at different concentrations of exogenous PDAP protein. Quantification was the percentage of cells positive for EdU. The mean ± SD of biological replicates is shown. Statistical analyses were performed using the Kruskal‐Wallis test (*p < 0.05; **p < 0.01; ***p < 0.001; *****p < 0.0001).

Cellular senescence is an important hallmark of human aging and longevity. We performed a series of experiments in vitro to verify the role of PDAP1 in cellular senescence as a potential target for longevity. We found that PDAP1 expression increases during cellular senescence across multiple models. We first constructed a replicative cellular senescence model by serial passage of lung primary fibroblast MRC5 cells as previously described (Mendez‐Bermudez et al. 2022). PDAP1 level was constant when cells did not undergo senescence (Figure 4A) and was upregulated after cells entered the pre‐senescence state at both transcription and protein levels (Figure 4A,B). This increased expression persists until the cells enter the senescence state. In addition, PDAP1 was also upregulated in stress‐induced cellular senescence models by ultraviolet radiation (Zhao et al. 2022a) (Figure 4C) and chemotherapeutic drug (Zumerle et al. 2024) (Figure 4D), which was consistent with our Mendelian randomization and genetic colocalization results.

We then investigated the effect of PDAP1 on senescence by exposing cells to varying concentrations of PDAP1 recombinant protein. We observed a dose‐dependent increase in the ratio of SA‐β‐gal‐positive MRC5 cells (the percentage of SA‐β‐gal‐stained positive cells to the total number of cells, which means the proportion of cells in the senescent state) following stimulation with exogenous PDAP1 (Figure 4E). The levels of p16 and p21, which are indicators of cellular senescence, also increased gradually with PDAP1 stimulation (Figure 4F,G). In contrast, the ratio of EdU‐positive cells (the percentage of EdU‐stained positive cells to the total number of cells, which means the proportion of cells in the proliferative state) dose‐dependently decreased with PDAP1 stimulation (Figure 4H).

### 2.9 PDAP1 Knockdown Extends Cellular Lifespan and Reduces Senescence Markers

**FIGURE 5:**
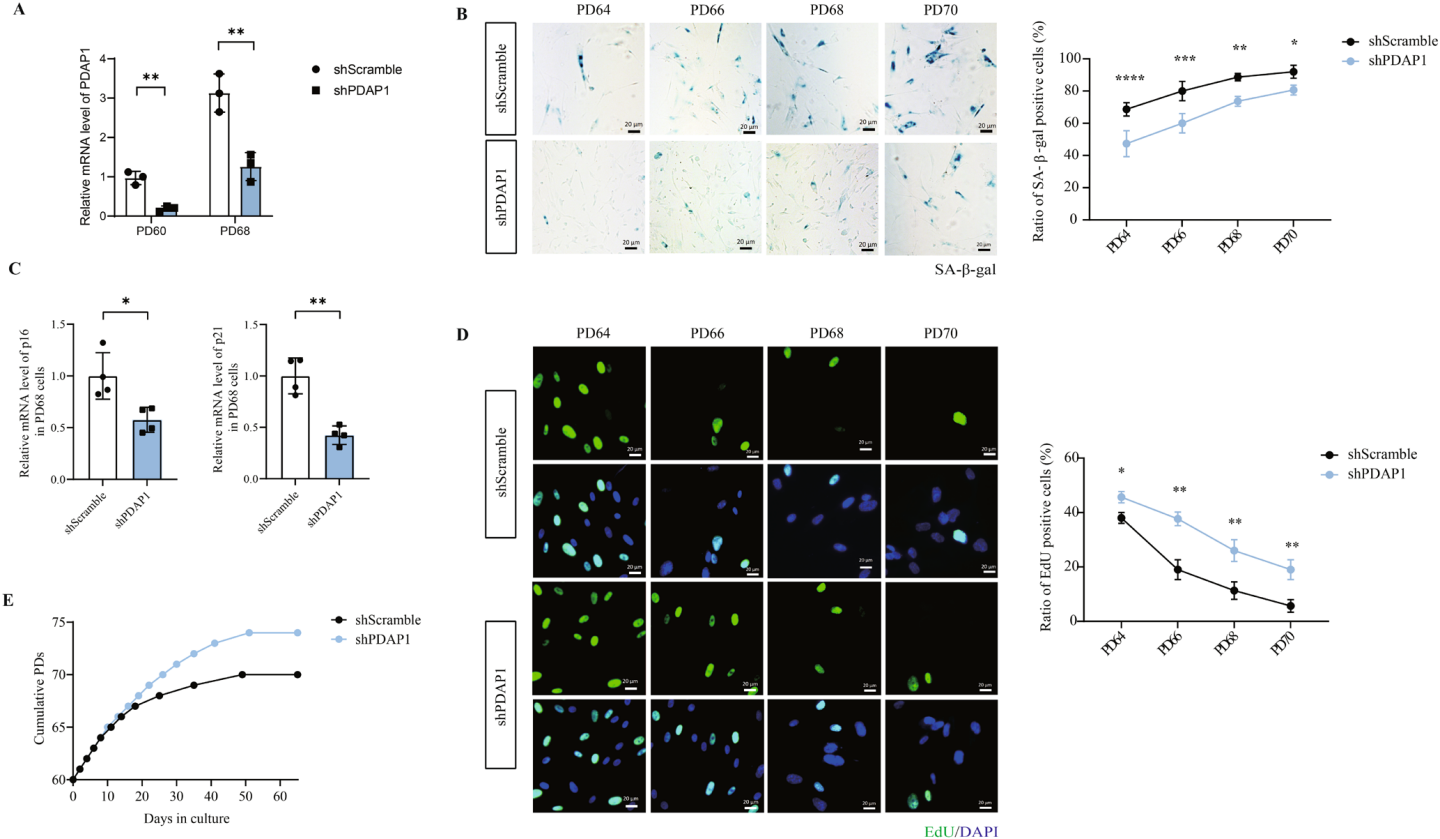
PDAP1 knockdown alleviates cellular senescence. (A) The mRNA expression levels of PDAP1 in MRC‐5 cells carrying either a vector expressing a scramble shRNA or PDAP1 shRNA. (B) Representative images showing the senescence‐associated β‐galactosidase in MRC‐5 cells at different population doublings (PDs) with or without PDAP1 silencing. Quantification was the percentage of cells positive for senescence‐associated β‐galactosidase. (C) Real‐time quantitative reverse transcription polymerase chain reaction analysis comparing p16 and p21 mRNA expression in PD 68 MRC‐5 cells with or without PDAP1 silencing. (D) Representative images showing the EdU in MRC‐5 cells at different population doublings (PDs) with or without PDAP1 silencing. Quantification was the percentage of cells positive for EdU. (E) Growth curve of MRC‐5 cells with or without PDAP1 silencing by lentivirus at PD 60. The cells were allowed to grow until senescence at 5% oxygen. The mean ± SD of biological replicates is shown. Statistical analyses were performed using the Kruskal‐Wallis test (*p < 0.05; **p < 0.01; ***p < 0.001; **** *p* < 0.0001).

To further answer whether targeting PDAP1 can delay cellular senescence, we downregulated PDAP1 levels in MRC5 cells from PD60 (60th population doubling) until the cells entered the senescent state by shRNA (Figure 5A). Compared with the control cells within the same PD, the ratio of SA‐β‐gal‐positive cells and the levels of p16 and p21 decreased in the PDAP1 downregulated group (Figure 5B,C), while the ratio of EdU‐positive cells increased (Figure 5D), and this difference continued until the control cells entered the senescent state. More importantly, during continuous subcultures, cells with downregulated PDAP1 exhibited stronger proliferation ability and extended the culture period of about 4PD before entering the senescent state compared with the control cells (Figure 5E).

## 3 Discussion

By integrating a large sample size of genetic data on tissue‐specific gene transcripts and circulating proteins, our analysis drew a comprehensive picture of the critical gene transcripts, plasma proteins, and the intermediate diseases or traits that possess putative causal effects on human longevity traits. We identified 14 serum proteins and 9 whole‐blood genetic transcripts that had causal effects on longevity outcomes. Four proteins, lipoprotein(a) (LPA), PDGFA‐associated protein 1 (PDAP1), DnaJ heat shock protein family (Hsp40) member A4 (DNAJA4), and Transmembrane protein 106B (TMEM106B), with their corresponding genetic transcripts across 6 tissues had a consistent causal effect on health longevity. Particularly, PDAP1 emerged as a potential functional and actionable target for modifying lifespan, affecting SHBG, adiposity, waist circumference, and epigenetic aging acceleration. In an independent cohort, the increasing plasma level of PDAP1 was significantly associated with higher all‐cause mortality and more years of loss. In vitro, we demonstrated that upregulation of PDAP1 promotes cellular senescence by increasing senescence markers and reducing cell proliferation, while its downregulation delayed the onset of senescence and extended cell lifespan. These findings have the potential to provide valuable insights into drug targets that can promote a healthy lifespan.

Previous observational studies have identified dozens of genetic targets associated with longevity, while our study provided evidence for an extensive list of proteins. In addition to previously confirmed proteins, such as lipoprotein (a) and NT‐proBNP, we identified PDAP1, DNAJA4, SCARF2, CTSB, and CTSF as potential novel causally longevity‐related targets (Hirata et al. 2020; Morris et al. 2019; Pietri and Stefanadis 2021). We also found nine genetic transcripts in the whole blood that had causal effects on human longevity, including *HYKK*, *NRG1*, *NTN5*, *BECN1*, *ADD1*, *PDAP1*, *SRFBP1*, *KALRN*, and *RP11‐71H17.7*, which were mostly novelly discovered. The phenome‐wide mediation MR analysis showed that these proteins and genetic transcripts affected human longevity partially through cardiopulmonary metabolic factors, including modifying lipids (high‐density lipoprotein) levels, lung function, basal metabolic rate, or influencing the pathogenesis of heart failure and hypertension.

Rather than investigating the transcripts/proteins separately, we have been able to establish concordance between plasma‐derived pQTLs and cell‐derived eQTLs through the utilization of data from plasma proteins and tissue/cell‐specific expression (Jamie et al. 2022). Following the central dogma of molecular biology, proteins are produced through the expression of genes based on individual DNA molecules within living cells. Our tissue‐specific analysis identified PDAP1 and DNAJA4 as novel driving targets for longevity derived from their respective tissues. We also found that specific tissues secreting the lifespan‐related proteins lipoprotein (a), PDAP1, DNAJA4, and TMEM106B had negative causal effects on longevity (Hirata et al. 2020; Morris et al. 2019; Napolioni et al. 2011). For instance, TMEM106B, as a progranulin protein, has previously been linked to aging in mice and *C. elegans* and in humans (Ahmed et al. 2010; Baker et al. 2006; Butler et al. 2019; Rhinn and Abeliovich 2017). Here, we provided evidence that both eQTLs and pQTLs of TMEM106B may decrease the possibility of reaching the top 1% extreme longevity by dementia. Interestingly, cross‐sectional studies showed that plasma levels of DNAJA4 increased with various types of cancers (Chatterjee and Burns 2017). At the same time, we found that genetic transcripts of DNAJA4 negatively influenced parental lifespan across the thyroid and skin. These tissue‐specific results suggest that targeting specific human organs may be a way to counteract aging.

Specifically, we found that both transcript and serum protein levels of PDAP1 were casually associated with human lifespan. PDAP1, also known as platelet‐derived growth factor subunit A‐associated protein 1, is a multi‐tissue‐expressed protein in the pathway that suppresses T‐cell function. It has become a potential target for *c‐myc* and contributes to carcinogenesis (Chirichella et al. 2022; Cui et al. 2022). We found that PDAP1 exhibited robust MR results with the highest posterior probability of both bi‐ and multi‐traits colocalization on lifespan. Mediation analysis suggested that PDAP1 decreased longevity via decreased SHBG, increased waist circumference, blood pressure, smoking, and alcohol consumption in addition to cancer. Studies also suggested that lower SHBG levels may increase testosterone levels, leading to cardiovascular risk factors (Aribas et al. 2021; Jiang et al. 2023; Sugrue et al. 2021). The harmful effects of altered diseases or traits were also consistent with the epigenetic acceleration of aging caused by PDAP1. These results were consistent with our findings from the UK Biobank, where the plasma level of PDAP1 was significantly associated with all‐cause mortality and life expectancy. In addition, the in vitro experiments indicated targeting PDAP1 offers a dual advantage by potentially promoting senescence in cancerous cells to inhibit growth, while delaying senescence in healthy cells to enhance tissue regeneration and extend cellular lifespan.

Our study possesses several strengths. Most importantly, we combined and consolidated evidence from pQTLs and eQTLs for drug target validation to ensure consistency and shared signals between the two molecules to minimize misleading results. In addition, we investigate the tissue specificity of QTLs in target validation applications to emphasize the concordance of eQTL and pQTL pairs across tissues in target validation. It is worth noting that when phenotype‐associated genetic variants are directly linked to gene expression levels and protein abundance, they are more likely to serve as successful drug targets (Jamie et al. 2022). This concordance could provide a potential highly bio‐functional target of longevity. We conducted a comprehensive phenome‐wide scan in an independent cohort and performed a mediation analysis to illustrate the potential mechanism linking QTLs to longevity. Finally, the use of multiple cellular senescence models, both replicative and stress‐induced, to validate the role of PDAP1, comprehensive mechanistic investigations through gain‐ and loss‐of‐function experiments, and the integration of MR and genetic colocalization analyses to strengthen the causal inference.

Several limitations should be acknowledged. First, the direction of some of the serum protein results contradicted the corresponding tissue‐specific eQTL results from GTEx. This could be due to the fact that the abundance of a plasma protein is more likely to correlate with the tissue that secretes the protein into plasma than with its expression in other tissues. Second, the proteomic MR was based on existing shared proteomic data, where some novel but rare serum longevity‐related factors, such as PF4 and Klotho, may need to be uncovered given the insufficient statistical power (Schroer et al. 2023). Third, the current colocalization tool assumes one independent signal for each gene at each locus for the GWAS and QTL results. Colocalization based on multiple causal variants may need to be considered. Another limitation is the inability to include newer biological aging ‘clocks,’ such as the MetaboHealth score, as these markers currently lack GWAS data, which restricts their use in our analyses (Bizzarri et al. 2022; Kuiper et al. 2023). Forth, given the limited number of the SNP for PDAP1, MR sensitivity analyses such as MR‐Egger and Cochran's Q might not be able to exam the pleiotropy of PDAP1. Therefore, we perform the cohort study and in vitro experiments to validate the biological effect of PDAP1. Fifth, in addition to cellular experiments, an in vivo animal aging model is still required to explore changes in mammalian lifespan after direct intervention of PDAP1 levels in plasma. Last, given the need for more data on both QTLs and longevity in other populations, the current study can only focus on the genetic targets in the European population.

In conclusion, this study utilized an integrated genetic approach to identify actionable genetic targets for enhancing human longevity. These findings represent a significant stride forward in identifying intervention targets that may ultimately increase lifespan and enable extreme longevity. In the future, these identified candidate proteins need to be validated and applied in prediction and molecular subgroups of major diseases in either larger prospective follow‐up cohorts or clinical studies. The candidate proteins, such as PDAP1, are warranted to further explore their molecular mechanism as potential targets for drug intervention.

## 4 Methods

### 4.1 Study Design and Participants

In this study (Figure 1), we aimed to prioritize potentially actionable targets for human lifespan and extreme longevity by integrating multi‐omics analysis methods and investigating the biological function of the filtered candidate targets. Plasma proteome and multi‐tissue transcriptome (GTEx Consortium 2020; Liu et al. 2022; Võsa et al. 2021) were used as exposures and the parental lifespan, and the top 1% and top 10% extreme longevity were used as outcomes (Deelen et al. 2019; Timmers et al. 2019). The putative causal effects of the selected genetic targets on longevity were estimated using MR and colocalization with several sensitivity analyses. A phenome‐wide mediation MR with PPI and enrichment analysis was conducted to investigate potential mechanisms linking eQTLs/pQTLs with longevity. Finally, we verified the potential longevity targets in the independent population from the UK Biobank and performed a series of cellular senescence experiments in vitro to prove the biological plausibility of the prioritized longevity targets. All the data sources are shown in Table S1.

### 4.2 Genetic Instruments for eQTL and pQTL Selection and Validation

We used a previously well‐defined pipeline to generate genetic instruments for potential genetic targets (Chen et al. 2022; Zheng et al. 2020). We extracted pQTLs for plasma proteins extracted from five genetic studies, the consortia of INTERVAL, KORA, the Atherosclerosis Risk in Communities (ARIC), the Framingham Heart Study (FHS), and a meta‐analysis of 90 plasma proteins (Folkersen et al. 2020; Suhre et al. 2017; Sun et al. 2018; Yao et al. 2018; Zhang et al. 2022) while pQTLs from an independent cohort, the DECODE study, as a validation. We extracted eQTLs of whole blood from the eQTLGen while tissue‐specific eQTLs from the top five expressed tissues of each gene were obtained from the GTEx V8 (GTEx Consortium 2020; Liu et al. 2022; Võsa et al. 2021).

Variants in the *cis*‐acting region of the protein‐coding loci or expression loci (±500 kb) that were significantly associated with plasma protein levels or gene transcript expression levels (*p* < 5 × 10^−8^) (*p* < 1 × 10^−5^ for GTEx due to limited sample size) after LD pruning (*r* ^2^ < 0.01) were used as primary instrument variables (IVs). To quantify the statistical power of eQTLs and pQTLs, we estimated the strength of the genetic predictors of each tested variant using F‐statistics. If any QTL had F‐statistics < 10, we considered those to have limited power (potentially causing weak instrument bias) and removed these from the subsequent analyses. Additionally, we used another two sets of IVs in the validation analysis following the same selection process: the variants in both the *cis*‐ and *trans*‐acting regions from the pQTLs and eQTLs, and the cis‐pQTLs from an independent cohort, the DECODE study.

To validate our primary results, we used another two sets of variants as additional analyses. First, we included variants in both *cis*‐ and *trans*‐regions that were significantly associated with either plasma proteins or gene transcripts under LD clumping (*r* ^2^ < 0.01). Second, we extracted *cis*‐pQTLs from an independent cohort, the DECODE study from Iceland, following the same variants selection process.

### 4.3 Longevity‐Related Outcome Selection

We included three outcomes representing human longevity: parental lifespan, top 1% extreme longevity, and top 10% extreme longevity. Longevity was defined as the top 10% survivors and beyond, which is able to be transmitted as a quantitative genetic trait (van den Berg et al. 2019). The GWAS with parental lifespan was obtained from a large meta‐analysis of parent survival in a sample of 1,012,240 parents (60% deceased) of European ancestry from UK Biobank and a previously published meta‐analysis of 26 additional population cohorts (Timmers et al. 2019). The GWASs for extreme longevity were from two meta‐analyses of 18 case–control studies in a European ancestry population: (1) age above the 99th percentile cases (N cases = 3484) and (2) age above the 90th percentile cases (N cases = 11,262), versus 25,483 controls whose age at death or last contact was at or below the age corresponding to the 60th survival percentile (Deelen et al. 2019).

To further understand the correlation between the three outcomes, we conducted a pairwise genetic correlation analysis of the three GWASs of longevity using bivariate LD score regression based on summary statistics, which calculates the cross‐product of test statistics at each variant and then regresses the cross‐product on the LD score (which is a measure of how much variation each variant tags). In total, three pairs of genetic correlations were estimated.

### 4.4 Mendelian Randomization and Sensitivity Analyses

In the discovery MR analysis, we estimated the putative causal effects of both gene expression and plasma protein levels in the *cis*‐acting region on the three longevity outcomes separately. For transcripts and/or proteins with only one instrument, we conducted Wald ratio analysis. For transcripts and/or proteins with two or more instruments, we applied the inverse variance weighted (IVW) approach (Hemani et al. 2018).

Given the potential violation of MR assumptions, we applied three sensitivity analyses for eQTLs/pQTLs that have two or more instruments. First, we applied the MR‐Egger method and considered the intercept term of the Egger approach as an indicator to estimate the potential effect of pleiotropy. For MR signals with an Egger intercept *p* < 0.05, we considered these signals as influenced by horizontal pleiotropy and excluded them in the following analyses. Second, we applied Cochran's *Q* test for IVW results to estimate the heterogeneity of MR estimates across each exposure‐outcome pair. Since heterogeneity could be caused by various reasons, we removed MR signals with evidence of heterogeneity (*P* in Cochran's *Q* < 0.05). Third, we used the MR‐PRESSO method to further exclude any results with undetected pleiotropy (Verbanck et al. 2018).

From a drug development point of view, a valid drug will influence the protein level, altering disease risk consequently (Zhao et al. 2022b). Therefore, we conducted a directionality test, Steiger filtering, to better understand the direction of effect of eQTL/pQTL‐longevity associations for all candidate MR signals. Any MR with Steiger filter flag as FALSE (which means the MR results explain more of the variance in the outcome than it does the variance in the exposure) was removed from the follow‐up analyses.

Since some proteins were measured repeatedly in different studies (INTERVAL, KORA, etc.), we conducted a subsequent meta‐analysis of MR results to consolidate distinct proteins into an integrated result based on the random‐effect model. All MR estimates after meta‐analysis with Benjamini‐Hochberg FDR corrected *p* < 0.05 were used to select candidate expression/protein‐longevity signals in follow‐up analyses.

### 4.5 Colocalization Analysis

For gene transcript or protein targets that showed robust MR effects on any one of three longevity outcomes in the discovery MR analysis, we conducted colocalization to distinguish causality from confounding by LD (Giambartolomei et al. 2014). This approach estimates the posterior probability of each genomic locus containing a single variant affecting both the target and the outcome. We used the default prior probabilities that a variant is equally associated with each outcome (*P*1 = 1 × 10^−4^; *P*2 = 1 × 10^−4^) and both outcomes jointly (*P*12 = 1 × 10^−5^). A posterior probability > 70% for the colocalization hypothesis in these analyses would suggest that the two association signals are likely to colocalize within the test region (noted as “*Colocalized*”).

### 4.6 Tissue Specificity Analysis

The plasma protein could be influenced or secreted by multiple tissues. Thus, we aimed to explore the effect of tissue‐specific gene transcription of candidate *cis*‐acting plasma proteins on human longevity. We extracted tissue‐specific eQTLs of significant plasma proteins based on the top 5 expressed tissues of the respective genes from GTEx V8 (GTEx Consortium 2020). We used genetic variants from significant gene pairs (*p* < 1 × 10^−5^) of selected *cis*‐acting plasma proteins with LD clumping of *r* ^2^ < 0.01 as the primary analysis. The same MR pipeline was applied for the tissue‐specific analysis. Significant tissue‐specific MR signals in the primary analysis with the same directional causality with the respective plasma proteins were finally included.

### 4.7 Multi‐Tissue Colocalization Analysis of Prioritized Targets

For candidate transcript and/or protein targets in MR, we explored whether the causal variants or regions were shared across transcriptome, proteome, and longevity outcomes. We employed a multi‐trait colocalization method implemented in the moloc R package (Giambartolomei et al. 2018). The default prior probabilities of 1 × 10^−4^ for any one layer of association, 1 × 10^−6^ for any two layers of associations, and 1 × 10^−7^ for colocalization of all three layers of associations were used in the moloc analysis (Detail in Supporting Information—Method S1). An overall colocalization probability of Pabc > 70% would suggest that the three traits shared one common causal variant and noted as “*Colocalized*” and an overall colocalization probability of three traits (*P*_a,b,c_ + *P*_a,bc_ + *P*_ab,c_ + *P*_ac,b_ + *P*_abc_) > 70% would suggest that the three association signals are likely to colocalize within the test region (“*Regional Colocalized*”).

### 4.8 Phenome‐Wide Association Study of Candidate Targets and Mediation MR Analysis

For all prioritized genetic transcripts and proteins with robust MR/colocalization evidence of lifespan, top 1% and top10% extreme longevity, we further conducted a phenome‐wide MR analysis (MR‐PheWAS) to identify potential beneficial and/or adverse mediators that connect these targets to longevity outcomes (Detail in Supporting Information—Method S2).

Both the pQTLs and eQTLs for the prioritized targets were chosen as the exposures for the MR‐PheWAS. For the potential mediating diseases or traits, we selected all available human diseases or traits in the IEU Open GWAS Project and performed a phenome‐wide MR of all phenotypes on an independent cohort from the UK Biobank, which included over 300,000 participants with parental age as a primary selection. The summary statistics of GWAS with the greatest expected statistical power, when multiple GWAS records of the same disease/risk factor were available, were selected. The MR process followed the same procedure as that for eQTL (instrument variables were selected after *P* for significance < 5 × 10^−8^ and LD clumping *r* ^2^ < 0.01 (3079 remained)). From all *P* values of IVW of all phenotypes on parental age in the UK Biobank < 0.1, we further determined 26 diseases and 40 traits as mediators for lifespan based on previous knowledge, where 10 diseases and 15 traits were further considered mediators for the top 1% and 10% extreme longevity.

### 4.9 Integration of Genetic and Biological Pathway Evidence

To better understand the causal mechanisms linking transcripts and proteins with human longevity, we further integrated the genetic evidence generated in this study with enrichment analysis and PPI information from the DAVID and STRING databases, respectively (Sherman et al. 2022) (Szklarczyk et al. 2023). To build a comprehensive genetic network, we selected candidate transcripts/proteins with all significant MR results for each outcome. We also performed a stepwise enrichment analysis of each subgroup of proteins clustered by K‐means in the PPI.

### 4.10 Observational Association Analysis in the UK Biobank

We then performed an observational study based on the UK Biobank to add population evidence to the target proteins. In the UKB, participants of the 2941 plasma protein analytes corresponding to 2923 unique proteins were measured using the antibody‐based Olink Explore 3072 proximity extension assay (PEA) technology. Protein measurements were expressed as normalized protein expression (NPX), a Log2 scale arbitrary unit. We searched the database for the genetically prioritized four proteins in Table 2; TMEM106B was not covered in the Olink Explore 3072 PEA panel. Thus, we included lipoprotein (α) and DNAJA4 as positive controls in adjustment model 4. In total, 46,799 randomly selected participants from the UK Biobank (*n* = 502,414) had available proteomics data.

Data for circulating levels of PDAP1, lipoprotein (α), and DNAJA4 were obtained from the UK Biobank. The circulating PDAP1 levels were defined as an exposure factor, both in continuous each‐unit increase and the categorical analysis to a tertile change (high‐, middle‐ and low‐group). We used multivariate Cox regression to assess the association of circulating levels of PDAP1 with all‐cause mortality based on 4 adjustment models: model 1, adjusted for age and sex; model 2 further adjusted for diabetes status, smoking status, and body mass index; model 3 further adjusted for systolic and diastolic blood pressure, total cholesterol, triglycerides, high‐ and low‐density lipoprotein cholesterol; model 4 further adjusted for protein Lp(a) and DNAJA4 levels. All results were expressed as the hazard ratio (HR) and 95% confidence interval (CI). First, residual life expectancy (i.e., years of life lost) was estimated as the area under the survival curve from age 45 up to 100 years (10‐year intervals) for participants with high‐ and middle‐circulating levels of PDAP1 with the low‐level group as reference. Years of life lost were calculated as the difference in life expectancy at any given age between participants with high‐ and middle‐circulating levels of PDAP1, respectively.

### 4.11 Cell Culture and Treatment

For the finally prioritized protein discovered in both genetic analysis and observational cohort (PDAP1), we performed basic cellular senescence experiments to explore its biological mechanism.

Lung primary fibroblast MRC5 cells were obtained from the ATCC. The MRC‐5 cells were cultured in DMEM supplemented with 10% fetal bovine serum (FBS) under a low‐oxygen environment (5% oxygen) until they reached senescence. Samples were then taken at different population doublings (PDs). The 3T3‐L1 cells were incubated for 48 h before being induced to differentiate into adipocytes with DMEM containing 10% FBS, 3‐isobutyl‐1‐methylxanthine (520 μM), dexamethasone (1 μM), rosiglitazone (1 μM) and insulin (6 μL/mL). After 48 h, the medium was replaced with DMEM containing 10% FBS and insulin (6 μL/mL) every 2 days for 5–7 days. All cells used in this study were maintained at 37°C and tested for mycoplasma contamination regularly. For UV‐irradiation treatment, cells were treated with a total energy of 10 J/cm^2^ UV irradiation by UV source. For doxorubicin and PDAP1 recombinant protein treatment, cells were starved overnight in a serum‐free medium and stimulated with 2 μM doxorubicin (MedChemExpress, HY‐15142) or varying concentrations of PDAP1 protein (Leading Biology, PH42259M5) for 12 h.

### 4.12 Senescence and Proliferation Assay

To evaluate cellular senescence, the senescence‐associated β‐galactosidase (SA‐β‐gal) activity in cultured cells was detected using the Senescence Detection Kit (Beyotime, C0602) according to the manufacturer's instructions. To evaluate cellular proliferation, EdU was added to the culture for 24 h at a 1 μM final concentration and detected using the Click‐iT EdU Alexa Fluor 647 Imaging Kit (Thermo Scientific, C10340). Cells were identified as being in a pre‐senescent state when the number of SA‐β‐gal‐positive cells began to increase and the number of EdU‐positive cells decreased. The fully senescent state was defined as all cells with SA‐β‐gal signals and EdU‐positive cells accounting for < 1%, as reported previously (Mendez‐Bermudez et al. 2022).

### 4.13 Lentivirus Infection

We used lentiviral vectors and short hairpin RNA (shRNA) to knock down endogenous PDAP1 levels. Lentiviruses were produced by transient PEI transfection of 293 T cells with the psPAX2 and pMD2.G virus packaging plasmids as well as the lentiviral expression vector that contained the sequence of interest. Titration was performed approximately 3 days after infection using puromycin (2 μg/mL) to select clones. The pLKO5‐GFP‐puro‐shPDAP1 (sh#1 CTTTCGAGGAGAGAACGAGAA; sh#2 GCTGCTATCTTTGAGACAGAA; sh#3 CGATGCCACATTGTCAGGAAA) was purchased from Sigma. The efficiency of shRNA was checked routinely by RT‐qPCR. The MRC‐5 cells for subsequent treatment were infected with lentiviruses in a growth medium containing 7 μg/mL polybrene and selected in 2 μg/mL puromycin for 7 days.

### 4.14 Western Blotting

Total protein extracts were obtained using ice‐cold RIPA buffer complemented with DMSF (Beyotime Biotechnology), phosphatase, and protease inhibitors (Roche) for 20 min. Protein concentration was quantified by PierceTM BCA Protein Assay Kit (Thermo). Protein was loaded onto SurePAGE 10% BisTris gradient gels (Genscript). The samples were transferred onto Immobilon‐P PVDF 0.45 μm membranes (Millipore) using a Trans‐Blot Turbo Transfer System (BioRad) followed by at least 1 h blocking with TBST in 5% skim milk. Hybridization with primary anti‐PDAP1 antibody (Thermo Scientific, PA5‐44938) was performed overnight at 4°C, followed by 1 h incubation with secondary horseradish peroxidase‐conjugated antibodies. Imaging was detected using AI600 (GE) and processed using ImageJ software.

### 4.15 Real‐Time qPCR

Total RNA was extracted using TRIzol reagent (Ambion) and reverse transcribed using the PrimeScript RT reagent kit (TaKaRa) according to the manufacturer's protocols. RT‐qPCR analysis was performed using the QuantStudio Dx Real‐Time PCR Instrument (ABI) with SuperRealPreMix Plus (TianGen). The primers used in this study are shown in the Supporting Information table of primers.

### 4.16 Analysis Software

The MR analyses were conducted using the “TwoSampleMR” R package. Colocalization analysis was conducted using the “coloc” and “moloc” packages. LD score regression was conducted using “LDSC” software. Enrichment analysis was performed using DAVID, and PPI analysis was performed based on the STRING database and Cytoscape 3.10. For the analysis in the UK Biobank, SAS version 9.4 (SAS Institute) and Stata (version 14) were used to perform the statistical analysis, and a two‐sided *p* < 0.05 was considered statistically significant differences. GraphPad Prism 7 software was used to generate experiment graphs and to perform statistical analysis. *P* values were obtained using either the two‐tailed Student's *t*‐test, Mann–Whitney *U* test, or the Kruskal‐Wallis test. Differences were considered statistically significant when *p* < 0.05 (*p < 0.05, **p < 0.01, ***p < 0.001, ****p < 0.0001).

### 4.17 Data Sharing

Data source was summarized in Figure 1 and Table S1. In detail, the eQTLs used as instruments were extracted from the eQTLGen (https://eqtlgen.org/), the GTEx (https://gtexportal.org/home/), and the Human Kidney Atlas (https://susztaklab.com/GWAS/). The pQTLs used as instruments were from the INTERVAL (from GWAS Catalog & IEU Open GWAS Project), the KORA (from IEU Open GWAS Project), the ARIC (http://nilanjanchatterjeelab.org/pwas/), the FHS (https://www.nature.com/articles/s41467‐018‐05512‐x), and data reported by Folkersen et al. (http://www.scallop‐consortium.com/). The GWAS summary data for parental lifespan was from Timmers et al. (https://datashare.ed.ac.uk/handle/10283/3209), and top 1% and 10% extreme longevity were from Deelen et al. (https://www.longevitygenomics.org/downloads). The UK Biobank individual level data (Application number: 70579), which are available from the UK Biobank (http://biobank.ndph.ox.ac.uk/showcase/). The code could be found in the Github (https://github.com/Timothy‐Hou/Longevity‐MR).

### 4.18 Ethic Statement

The genetic analyses of the present study only used publicly available summary‐level statistics. Ethical approval is therefore not required. The cohort analyses of the present study were based on the UK Biobank. All participants voluntarily provided written consent for their involvement in the study, and the research has received approval from the North West Multi‐Center Research Ethics Committee (Manchester, U.K.).

## Author Contributions

M.X., T.H., Z.S., and Q.W. conceived and designed the study, provided the methodology, formal analysis, and investigation of the study, wrote the original draft, and reviewed and edited it. J.Z., J.Y., W.W., Y.B., and W.H. reviewed and edited the manuscript. G.N., Y.B., and M.X. provided resources, reviewed and edited the manuscript, and acquired funding. All authors contributed to the interpretation of data, proofreading of the manuscript for important intellectual content, and gave final approval of the version to be published. M.X. is the guarantor for this study. The corresponding authors attest that all listed authors meet authorship criteria and that no others meeting the criteria have been omitted.

## Conflicts of Interest

The authors declare no conflicts of interest.

## Supporting information


Data S1.

Data S2.

## Data Availability

Data sharing is not applicable to this article as no new data were created or analyzed in this study.
