# Supplementary material for: Identifying PDAP1 as a Biological Target on Human Longevity: Integration of Mendelian Randomization, Cohort, and Cell Experiments Validation Study
Source: Aging Cell. 2025 Apr 10;24(7):e70065. doi: 10.1111/acel.70065 (PMC12266772; doi:10.1111/acel.70065)
Supplement: Supplementary file 1 — Data S1. [file ACEL-24-e70065-s001.docx]

Online-Only Supplementary Material

Title: **Identifying PDAP1 as a biological target on human longevity: integration of Mendelian randomization, cohort, and cell experiments validation study**

Tianzhichao Hou, Zimo Sha, Qi Wang, Yuanyue Zhu, Zheng Zhu, Huajie Dai, Yijie Zhu, Tiange Wang, Mian Li, Zhiyun Zhao, Yu Xu, Jieli Lu, Jie Zheng, Jing Ye, Weiqing Wang, Guang Ning, Yufang Bi, Weiguo Hu, Min Xu

**Supplementary Figures**

Supplementary Figure 1. Venn graph for cis-acting pQTLs on longevity outcomes.

Supplementary Figure 2. Protein-protein interaction (PPI) network for significant causal plasma proteins (pQTLs) on longevity outcomes.

Supplementary Figure 3. Enrichment analysis for cis-acting plasma proteins on human lifespan.

Supplementary Figure 4. Venn graph for cis-acting eQTLs on longevity outcomes.

Supplementary Figure 5. Manhattan plot for cis-eQTLs on longevity outcomes.

Supplementary Figure 6. Protein-protein interaction (PPI) network for significant causal transcripts (eQTLs) on longevity outcomes.

**Supplementary Methods**

Supplementary Method 1. Detail and methods of mediator selection.

Supplementary Method 2. Detail and methods of multi-traits colocalization.

**Supplementary Figure 1. Venn graph for cis-acting pQTLs on longevity outcomes.**


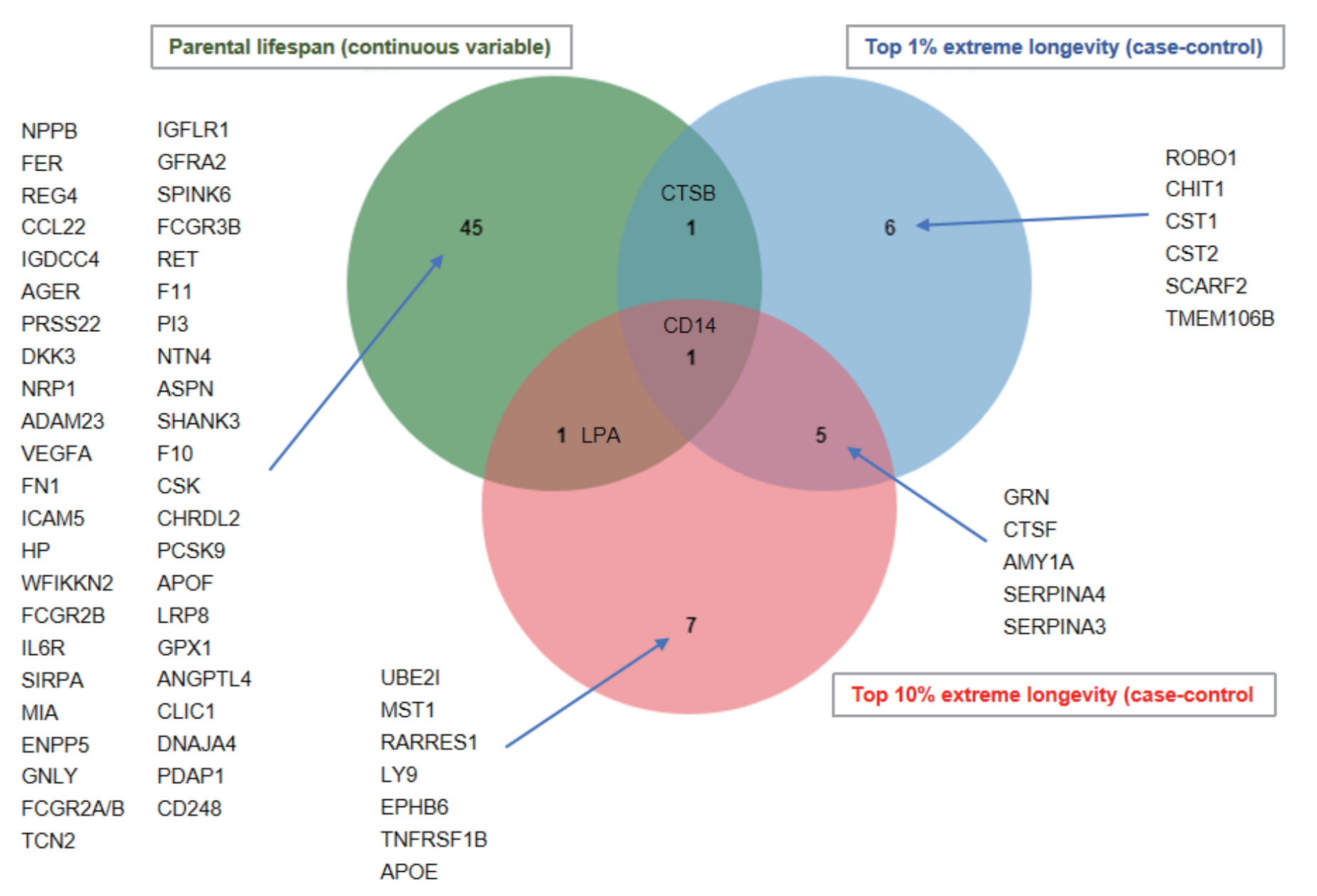


Note: The collection of MR signals of cis-acting pQTL after meta-analysis on top 10% extreme longevity (red circle), top 1% extreme longevity (blue circle), and lifespan (green circle). Under the threshold of FDR *q* <0.05, 5 cis-acting proteins overlapped between the top 1% and top 10% extreme longevity; 1 and 1 cis-acting proteins overlapped between lifespan and either the top 1% or top 10% extreme longevity, respectively. CD14 is the only significant causal proteins shared among the three outcomes after FDR correction.

**Supplementary Figure 2. Protein-protein interaction (PPI) network for significant causal plasma proteins (pQTLs) on longevity outcomes.**


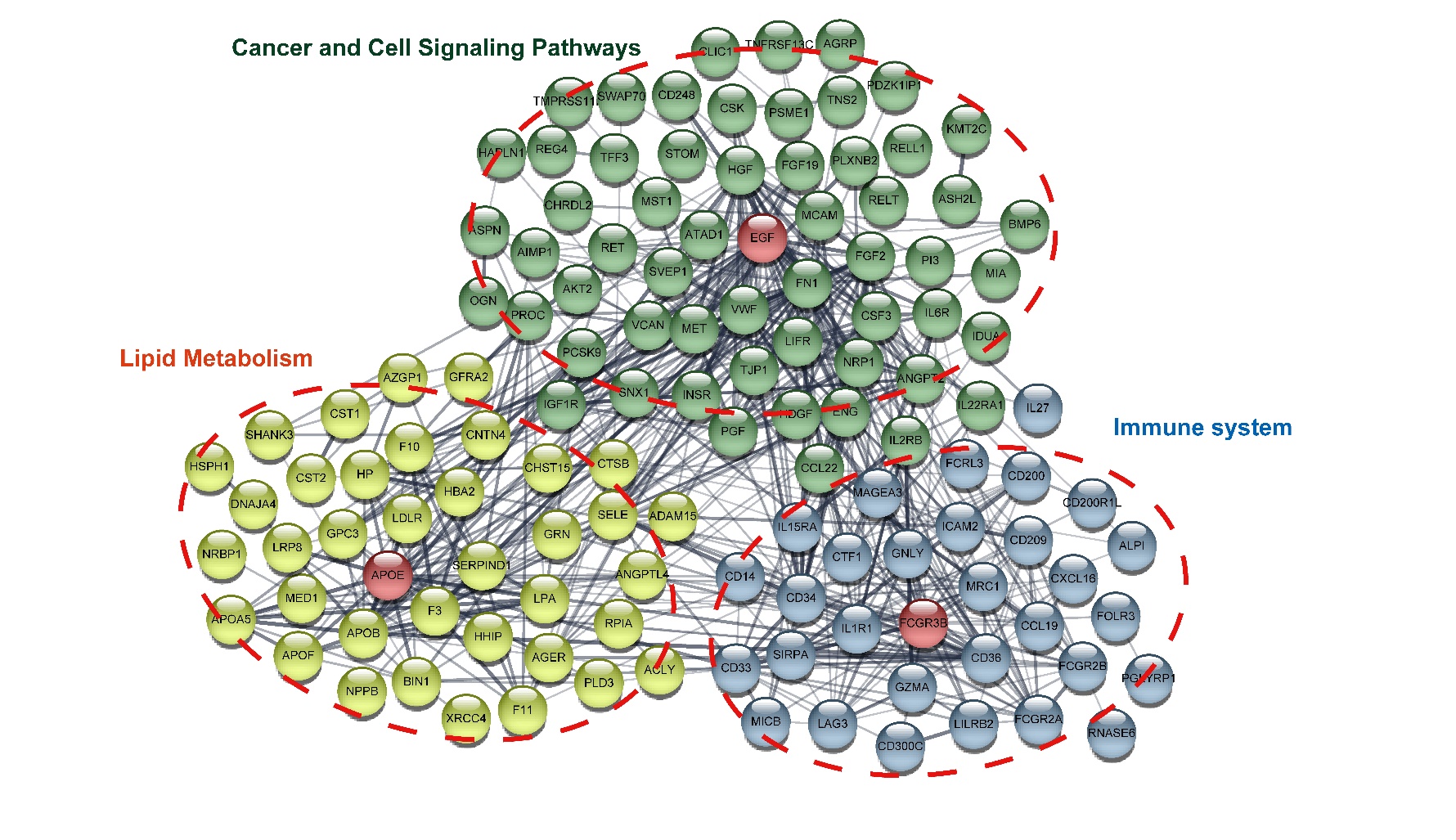


Note: A protein-protein interaction network with K-means clustering divided cis- and trans-acting proteins into neoplasm (green), immune response (blue), and lipid metabolism (yellow), which were verified in subgroup enrichment analysis. Each node represents one gene and only genes with previously known interaction (defined by STRING database) were kept in the figure. Genes without previously known interaction were removed.

**Supplementary Figure 3. Enrichment analysis for cis-acting plasma proteins on human lifespan.**

1. Enrichment analysis for the protein cluster of neoplasm (FDR q<0.05).


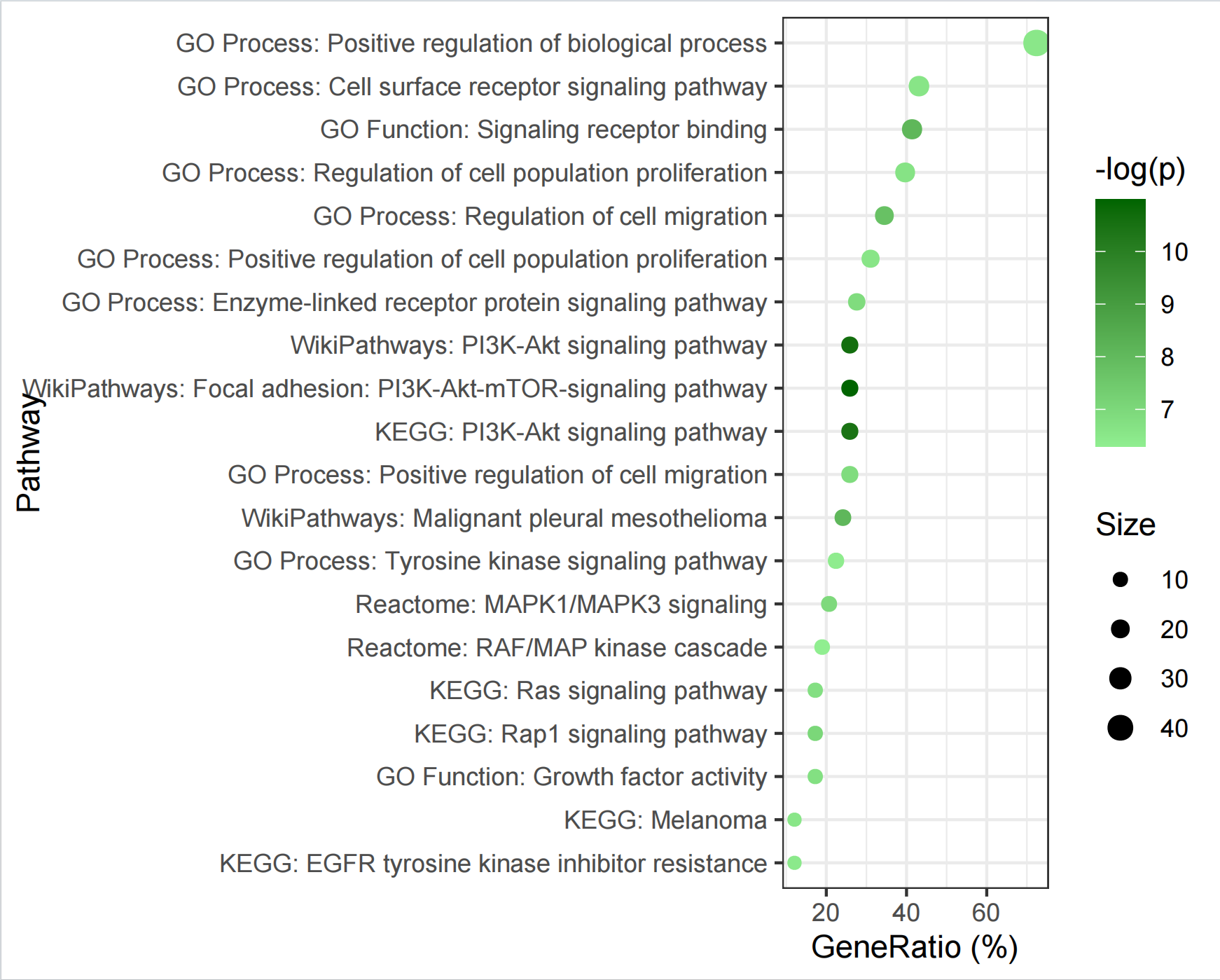


1. Enrichment analysis for the protein cluster of immune response (FDR q<0.05).


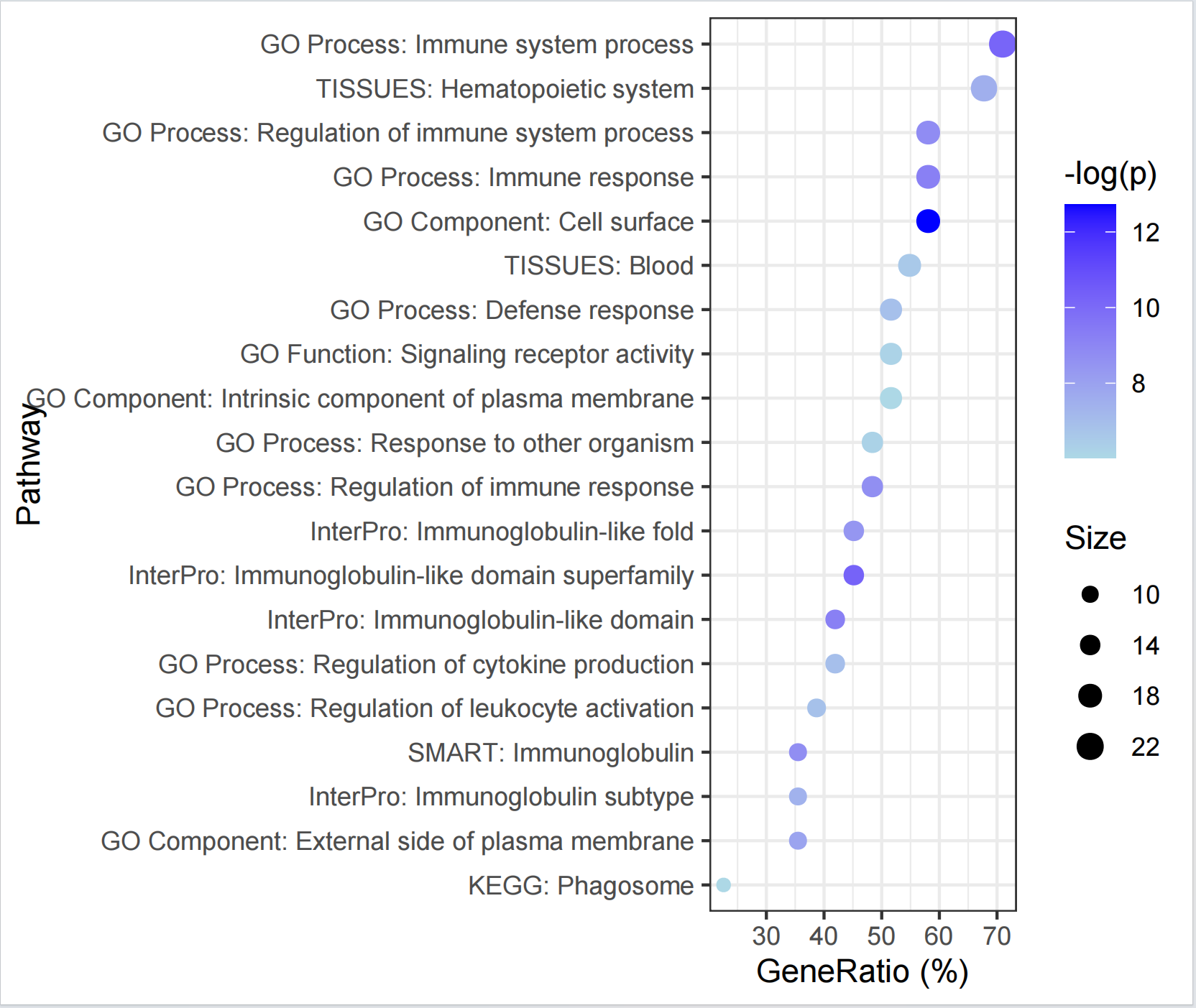


1. Enrichment analysis for the protein cluster of lipid response (FDR q<0.05).


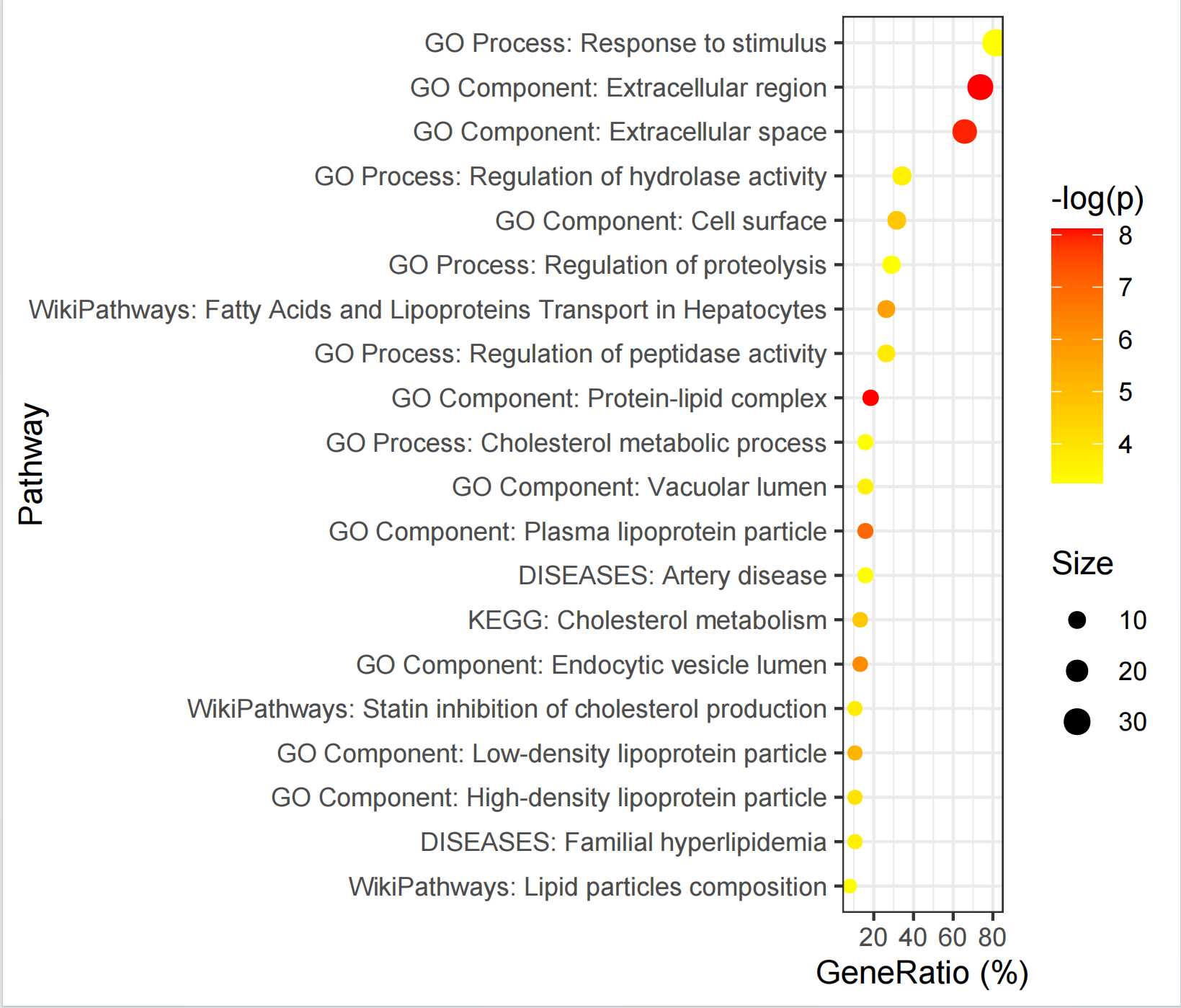


Note: All the shown components and pathways in each enrichment analysis reach significance after FDR correction.

**Supplementary Figure 4. Venn graph for cis-acting eQTLs on longevity outcomes.**


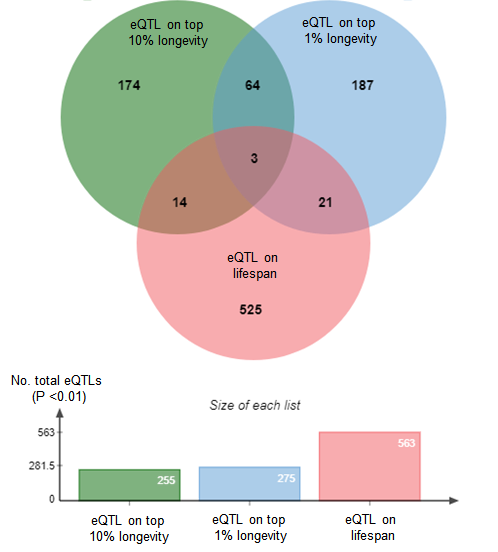


Note: The collection of MR signals (under a loose threshold: *P* <0.01) of cis-acting eQTL on top 10% extreme longevity (green circle), top 1% extreme longevity (blue circle), and lifespan (red circle). Under the threshold of *P* <0.01, 67 cis-transcripts overlapped between the top 1% and top 10% extreme longevity; 17 and 24 cis-transcripts overlapped between lifespan and either the top 1% or top 10% extreme longevity, respectively. No common cis_eQTLs from the three outcomes were found after FDR correction. Under the loose threshold of *P* <0.01, three cis-transcripts had a consistent causal effect on all three longevity outcomes: *WNT3*, *SYT11*, and *APEH*.

**Supplementary Figure 5. Manhattan plot for cis-eQTLs on longevity outcomes (GWAS suggestive threshold: *P* < 1× 10^-4^).**

1. Lifespan.


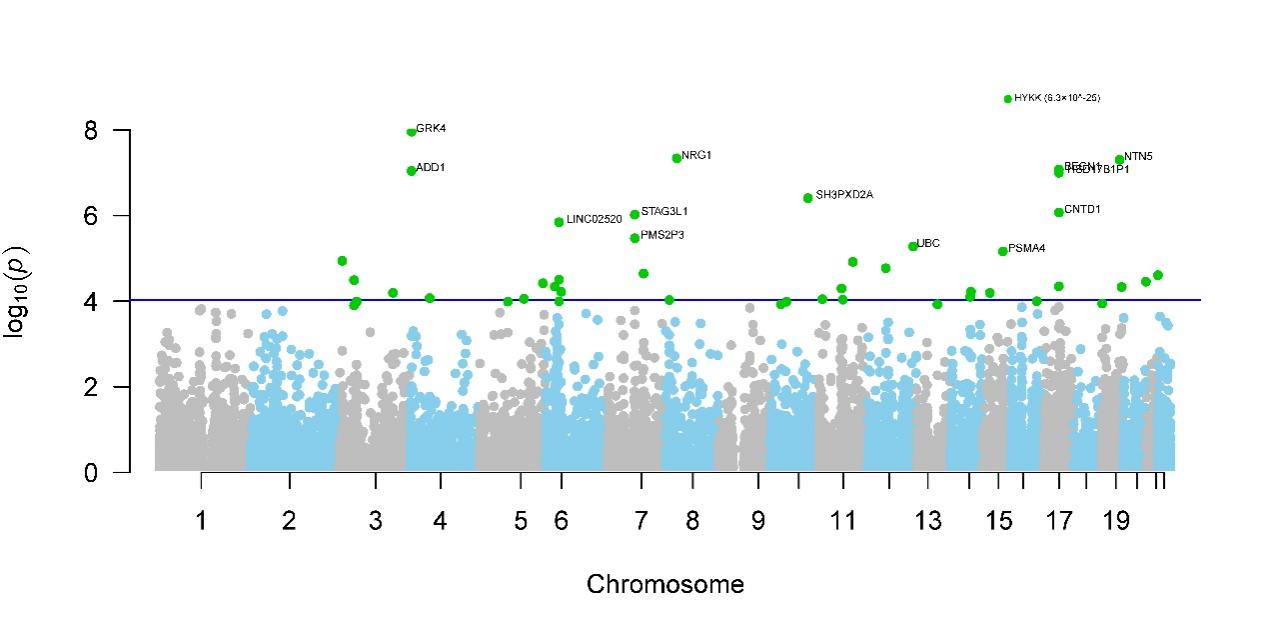


1. Top 1% extreme longevity.


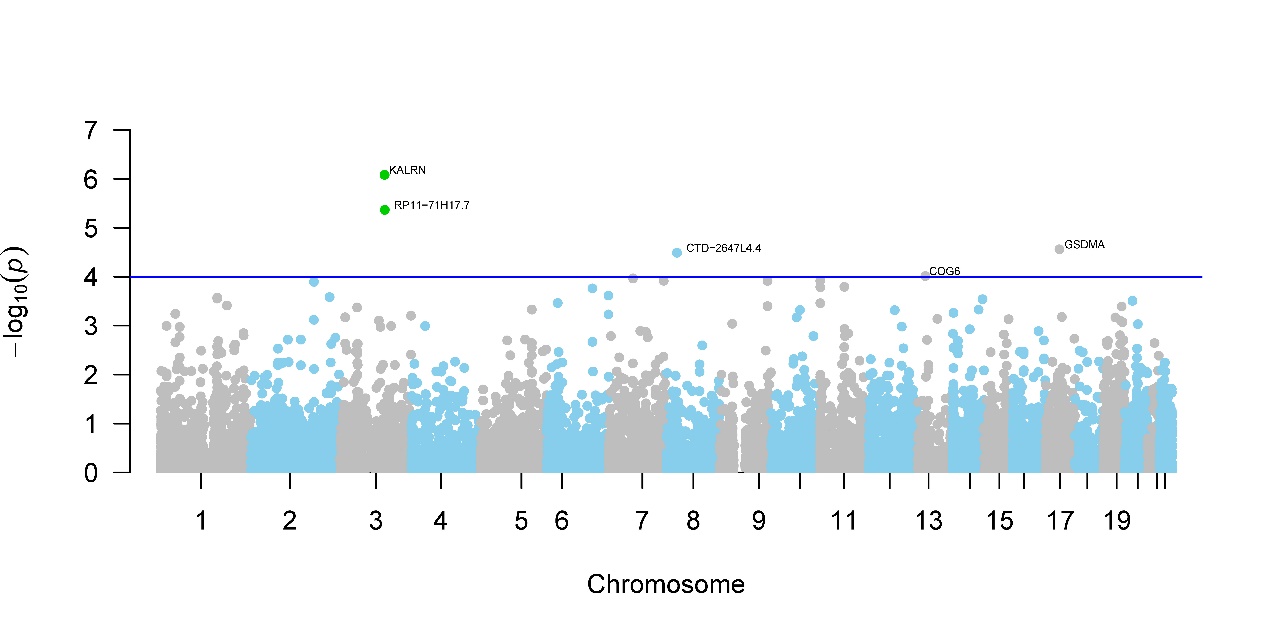


1. Top 10% extreme longevity


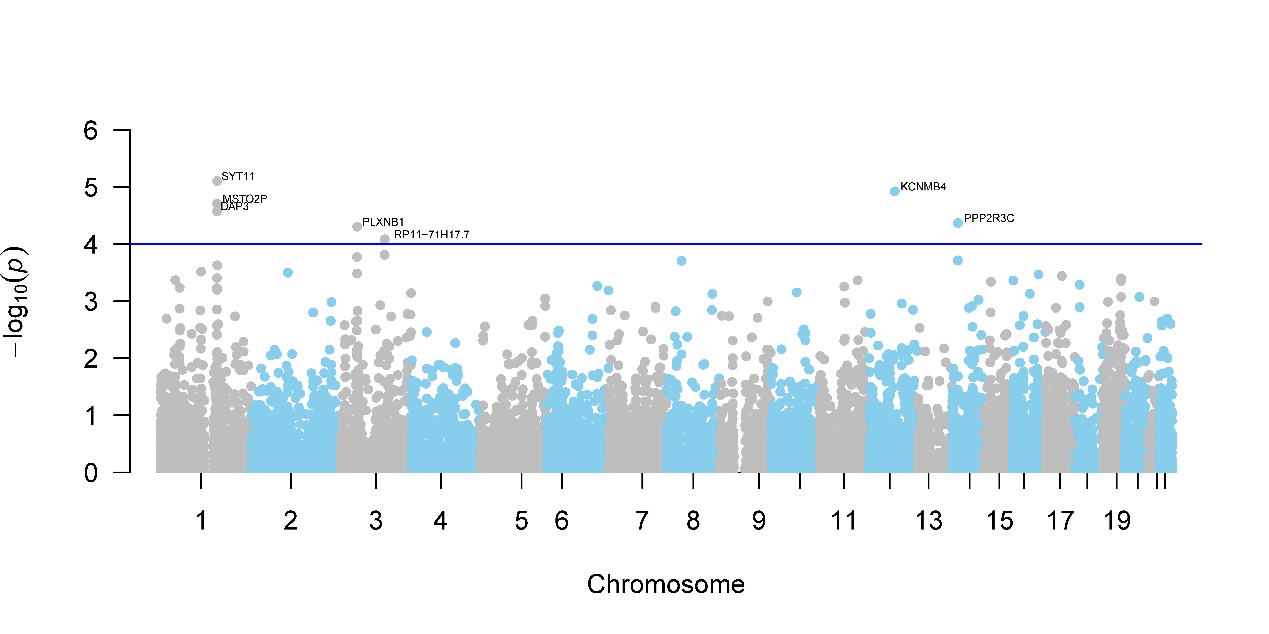


1. Comparison of Manhattan plot for cis-eQTLs on three longevity outcomes.


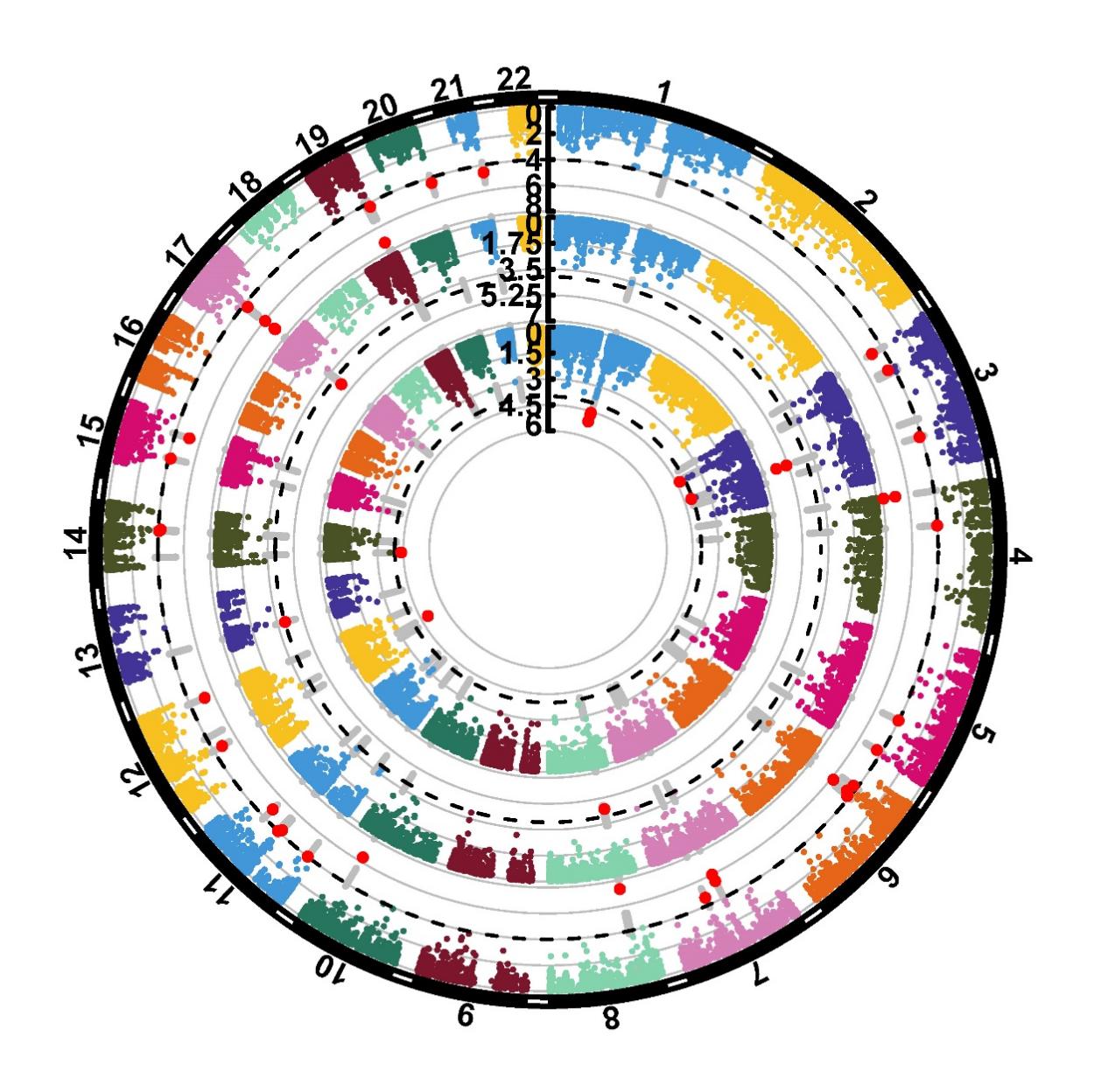


Note: A-C shows the genetic transcripts using cis-eQTLs on longevity outcomes (lifespan, top 1% extreme longevity, and top 10% extreme longevity). Blue lines indicate a suggestive threshold of *P* = 1× 10^-4^, which is a loose threshold commonly in all three outcomes for comparison (No significant cis-eQTLs in top 10% longevity if under FDR correction threshold). Green points are significant genetic transcripts after FDR correction. D. shows the comparison of Manhattan plot for cis-eQTLs on the three longevity outcomes. Outer circle indicates the lifespan; middle circle, top 1% extreme longevity, and inner circle, the top 10% extreme longevity. Red points are significant after the respective FDR correction. The middle line with scaled number is the -log_10_(P) values. The *HYKK* on chromosome 15 was not shown in the outer circle due to the largely difference of *P* value (6.25×10^-25^). **Supplementary Figure 6. Protein-protein interaction (PPI) network for significant causal transcripts (eQTLs) on longevity outcomes.**

A. PPI network for significant causal transcripts (eQTLs). Each node represents one gene and only genes with previously known interaction (defined by STRING database) were kept in the figure. Genes without previously known interaction were removed.


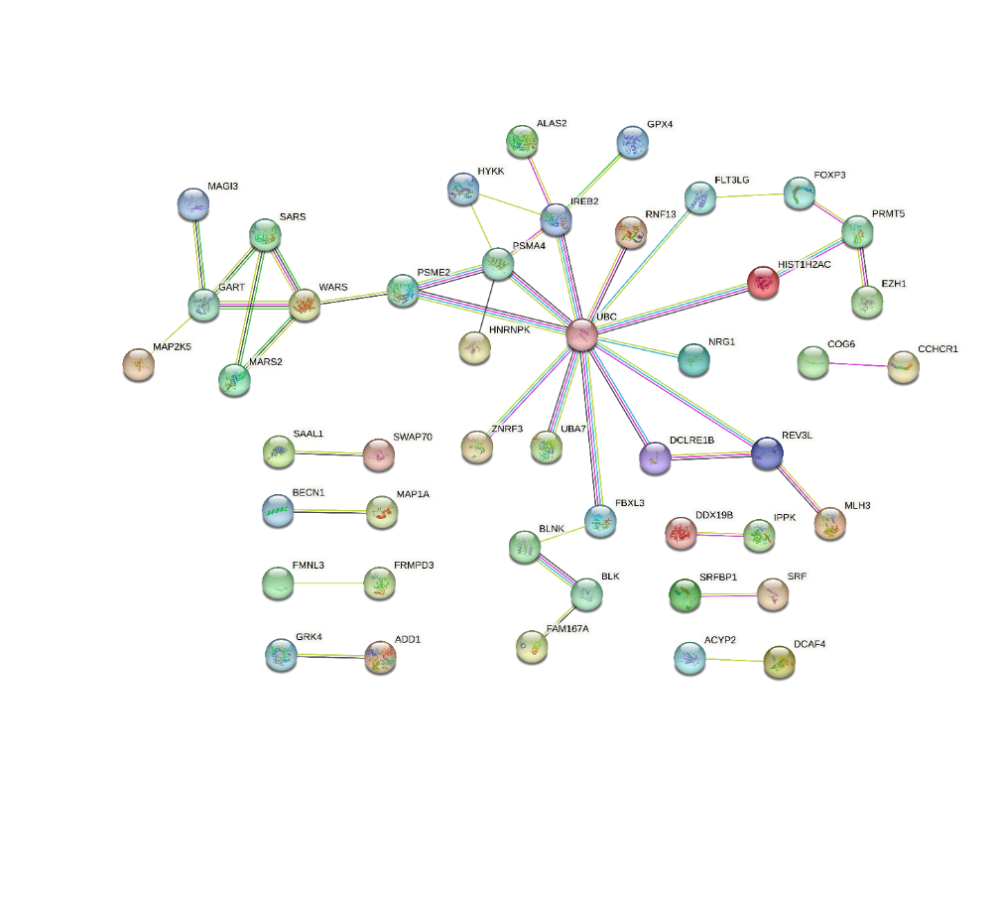


B. Original PPI network for ALL (both genes with/without previously known interaction in the STRING database) significant causal transcripts (eQTLs).


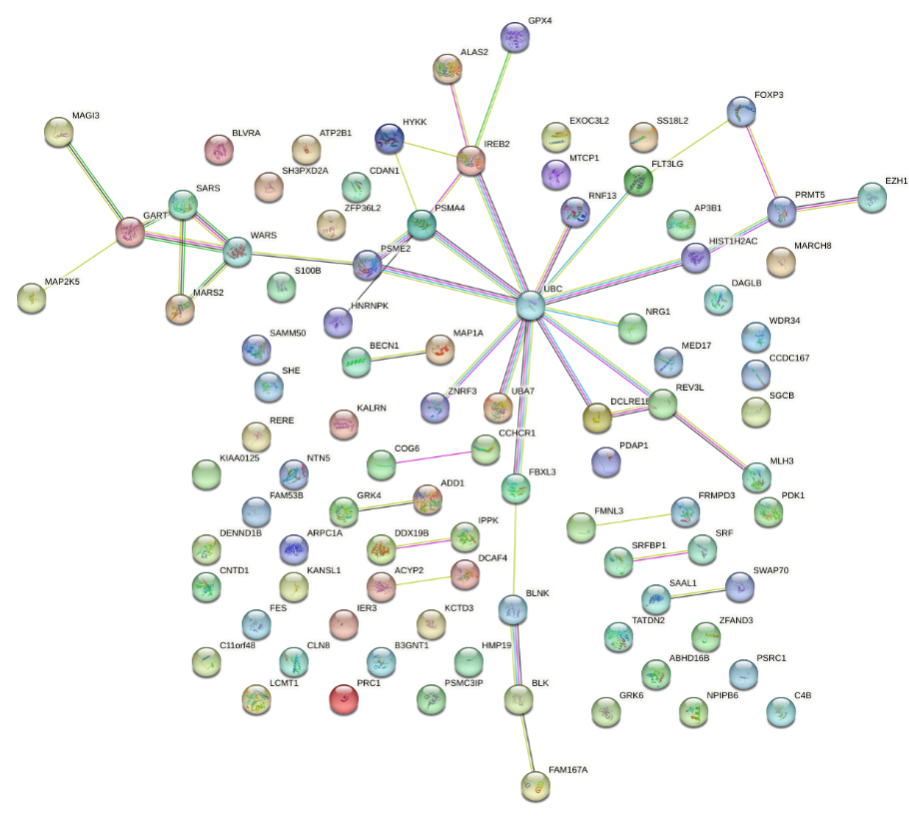


**Supplementary Method 1. Detail and methods of multi-traits colocalization.**

The multi-traits colocalization is a method for colocalization analyses among 3 traits. Here in our manuscript, this represents tissue-specific genetic expression (eQTL), plasma protein (pQTL) and longevity outcomes.

For three traits, the possible combinations of causal configurations (binary vectors with at most 1 entry equal to 1) can be assigned to one of the following 15 hypotheses listed below (rather 5 assumptions in the traditional 2 traits colocalization). Each hypothesis can be represented by a set of index set containing the traits each causal

SNP associates with.

- 1. H0 {φ}: No association in any trait.
  2. H1 {{1}}: One SNP is associated with trait 1 (a).
  3. H2 {{2}}: One SNP is associated with trait 2 (b).
  4. H3 {{3}}: One SNP is associated with trait 3 (c).
  5. H4 {{1, 2}}: One SNP is associated with trait 1 and 2 (ab).
  6. H5 {{2, 3}}: One SNP is associated with trait 2 and 3 (bc).
  7. H6 {{1, 3}}: One SNP is associated with trait 1 and 3 (ac).
  8. H7 {{1} , {2}}: One SNP is associated with trait 1, and a different SNP is associated with trait 2 (a.b).
  9. H8 {{2} , {3}}: One SNP is associated with trait 2, and a different SNP is associated with trait 3 (b.c).
  10. H9 {{1} , {3}}: One SNP is associated with trait 1, and a different SNP is associated with trait 3 (a.c).
  11. H10 {{1} , {2, 3}}: One SNP is associated with trait 1, and a different SNP is associated with trait 2 and 3 (a.bc).
  12. H11 {{3} , {1, 2}}: One SNP is associated with trait 3, and a different SNP is associated with trait 1 and 2 (c.ab).
  13. H12 {{2} , {1, 3}}: One SNP is associated with trait 2, and a different SNP is associated with trait 1 and 3 (b.ac).
  14. H13 {{1} , {2} , {3}}: One SNP is associated with trait 1, a different SNP is associated with trait 2, and a different SNP is associated with trait 3 (a.b.c).
  15. **H14 7 {{1, 2, 3}}: One SNP is associated with trait 1 and 2 and 3 (abc).**

The **Pabc** would indicate the posterior probability of three phenotypes shared a common significant causal SNP, and noted as “*Colocalized*”. An overall colocalization probability of three traits (Pa,b,c+Pa,bc+Pab,c+Pac,b+Pabc) >70% would suggest that the three association signals are likely to colocalize within the test region (“*Regional Colocalized*”).

**Supplementary Method 2. Detail and methods of mediator selection.**

The phenome-wide data were selected as potential mediating phenotypes between the molecular targets and longevity (mainly based on UK Biobank, FinnGen, and MRC-IEU).

1. **Available phenotypes filtration**: A total of 3079 phenotypes were selected from the IEU Open GWAS Project Platform with all available over 40,000 phenotypes. We kept health-related diseases and traits only while we excluded plasma proteins, genetic expressions, and phenotypes without certain definition. We also removed the duplicated phenotypes and kept the largest cohort only.
2. **Pre-determination of potentially longevity-related phenotypes**: We performed a phenome-wide MR of all 3079 phenotypes on a longevity outcome. This outcome was an independent cohort from the UK Biobank, which included over 300,000 participants with parental age, as a primary selection. Based on both a loose threshold of significance (*P* <0.1) and prior knowledge, we pre-determined **66 phenotypes** that were potentially causally associated with longevity outcomes.
3. **Verification and final determination of longevity-related phenotypes**: We performed MR analyses for the 66 phenotypes on our three longevity outcomes: parental lifespan, top 1% and top 10% extreme longevity. We finally **selected 26 health-related diseases and 40 traits** as mediators for **lifespan** (**Table S4**). Additionally, **10 health-related diseases and 15 traits** were considered as mediators for both the **top 1% and 10% extreme longevity** (**Table S5**).

**Mediation analyses**: For all prioritized genetic transcripts and proteins with robust MR/colocalization evidence of lifespan, top 1% and top10% extreme longevity, we further conducted a phenome-wide MR analysis (MR-PheWAS) to identify potential beneficial and/or adverse mediators that connect these targets to longevity outcomes.
